# NeuroEPO plus (NeuralCIM^®^) in mild-to-moderate Alzheimer’s clinical syndrome: the ATHENEA randomized clinical trial

**DOI:** 10.1186/s13195-023-01356-w

**Published:** 2023-12-13

**Authors:** Saily Sosa, Giosmany Bringas, Nelky Urrutia, Ana Ivis Peñalver, Danay López, Evelio González, Ana Fernández, Zenaida Milagros Hernández, Ariel Viña, Yamile Peña, Juan Felipe Batista, Carmen Valenzuela, Kalet León, Tania Crombet, Teresita Rodríguez, Leslie Pérez, on behalf of the ATHENEA Investigators, Yolanda Álvarez, Madelín Rodríguez, Nairim Vázquez, Mirelys Rodríguez, Yaniuris González, María A. Ramos, Yosvany López, Mara Hernández, Lázaro Madruga, Dianelys Carmona, Julio E. Acosta, Miriam López, Deiry Amaro, Olga L. Baños, Mariela Ortega Álvarez, Anay Cordero, Melany Betancourt, Liana Padrón, Elio Chávez, Isabel García, Yaquelin Morgan, Moraima Charles, Mónica González, Marianela de la C. Rodríguez, Yeniley León, Joe Michel López, Yanelis Acosta, Trinidad de los Ángeles Virués, Laura Pérez, Karen León, Rubén Periche, Adonisbel Valero, Yoelvis César Pozo, Greysi Horta, Rodobaldo Quesada, Elvia Luz, Leonel A. Torres, Susana Romero, María E. Rodríguez, Daymys Estévez

**Affiliations:** 1 Hospital Iván Portuondo, Calle 78 e/ Ave. 33 y 37, San Antonio de los Baños, Artemisa CP 32 500 Cuba; 2 National Institute of Neurology (INN), Calle 29 esquina D, Vedado, Havana CP 10 400 Cuba; 3 Cuban Neurosciences Center (CNEURO), Avenida 25, No. 15 007, Cubanacán, Havana CP 11 600 Cuba; 4 Center of Neurological Restoration (CIREN), Calle 216 esquina 13, Siboney, Playa, Havana CP 11 600 Cuba; 5 Center for Clinical Investigation, CENTIS, Calle 45 No. 4501, esquina a 34, Reparto Kolhy, Havana CP 11 300 Cuba; 6 Institute of Cybernetics, Mathematics and Physics (ICIMAF), Calle 15 #551 entre C y D, Plaza de la Revolución, Vedado, Havana CP 10 400 Cuba; 7 Center of Molecular Immunology (CIM), Calle 216 esquina 15, Siboney, Playa , Havana CP 11 600 Cuba

**Keywords:** Randomized controlled trial, NeuroEPO, Alzheimer’s disease, Neuroprotective

## Abstract

**Background:**

NeuroEPO plus is a recombinant human erythropoietin without erythropoietic activity and shorter plasma half-life due to its low sialic acid content. NeuroEPO plus prevents oxidative damage, neuroinflammation, apoptosis and cognitive deficit in an Alzheimer’s disease (AD) models. The aim of this study was to assess efficacy and safety of neuroEPO plus.

**Methods:**

This was a double-blind, randomized, placebo-controlled, phase 2–3 trial involving participants ≥ 50 years of age with mild-to-moderate AD clinical syndrome. Participants were randomized in a 1:1:1 ratio to receive 0.5 or 1.0 mg of neuroEPO plus or placebo intranasally 3 times/week for 48 weeks. The primary outcome was change in the 11-item cognitive subscale of the AD Assessment Scale (ADAS-Cog11) score from baseline to 48 weeks (range, 0 to 70; higher scores indicate greater impairment). Secondary outcomes included CIBIC+, GDS, MoCA, NPI, Activities of Daily Living Scales, cerebral perfusion, and hippocampal volume.

**Results:**

A total of 174 participants were enrolled and 170 were treated (57 in neuroEPO plus 0.5 mg, 56 in neuroEPO plus 1.0 mg and 57 in placebo group). Mean age, 74.0 years; 121 (71.2%) women and 85% completed the trial. The median change in ADAS-Cog11 score at 48 weeks was −3.0 (95% CI, −4.3 to −1.7) in the 0.5 mg neuroEPO plus group, −4.0 (95% CI, −5.9 to −2.1) in the 1.0 mg neuroEPO plus group and 4.0 (95% CI, 1.9 to 6.1) in the placebo group. The difference of neuroEPO plus 0.5 mg vs. placebo was 7.0 points (95% CI, 4.5–9.5) *P* = 0.000 and between the neuroEPO plus 1.0 mg vs. placebo was 8.0 points (95% CI, 5.2–10.8) *P* = 0.000. NeuroEPO plus treatment induced a statistically significant improvement in some of clinical secondary outcomes vs. placebo including CIBIC+, GDS, MoCA, NPI, and the brain perfusion.

**Conclusions:**

Among participants with mild-moderate Alzheimer’s disease clinical syndrome, neuroEPO plus improved the cognitive evaluation at 48 weeks, with a very good safety profile. Larger trials are warranted to determine the efficacy and safety of neuroEPO plus in Alzheimer’s disease.

**Trial registration:**

https://rpcec.sld.cu Identifier: RPCEC00000232.

**Supplementary Information:**

The online version contains supplementary material available at 10.1186/s13195-023-01356-w.

## Introduction

Alzheimer’s disease (AD) is a progressive neurodegenerative disorder and the most common form of dementia. Considerable research efforts have been directed towards developing safe and effective pharmacological treatments. The U.S. Food and Drug Administration (FDA) has approved seven drugs for the treatment of Alzheimer’s: rivastigmine, galantamine, donepezil, memantine, memantine plus donepezil, aducanumab (under the accelerated approval pathway), and lecanemab [1–7].

Lecanemab, the last drug approved by the FDA, reduced markers of amyloid in early Alzheimer’s disease and resulted in moderately less decline on measures of cognition and function than placebo at 18 months according Clarity AD trial [3, 4]. Another $\beta$-amyloid-targeting antibody: donanemab, slowed clinical progression at 76 weeks in early symptomatic Alzheimer’s, according to the TRAILBLAZER-ALZ 2 randomized clinical trial [8]. Thus, there is an urgent need for developing new therapies for preventing, delaying onset, slowing progression, and improving symptoms of AD, with good safety profile.

Erythropoietin (EPO) is a growth factor mainly produced in the kidney and with a well-known activity on erythropoiesis. The EPO receptor (EPOR) is differentially expressed in neurons, astrocytes, and endothelial cells in different regions of the central nervous system (CNS) [9]. Different functions of EPO in the CNS with respect to the hematopoietic system are most likely due to the four different isoforms of the EPOR, which are expressed specifically in different tissues. The canonical EPOR isoform present in the hematopoietic system enables erythroid differentiation, whereas in the brain it regulates neuroinflammation and hypoxia. The EPOR/$\beta$ cR (CD131) isoform is involved in neural tissue protection [10].

EPO binding to its cell surface receptor leads to a decrease in apoptosis, oxidative stress, and neuroinflammation. Studies have shown that EPO protects hippocampal and cortical neurons from glutamate-induced cell death in vitro and in vivo [11], as well as from A $\beta$ toxicity [12] by reducing inflammation [13] and by acting as an antioxidant [14]. Pre-clinical animal studies have shown that EPO improves neurological function [9, 15].

When administered intravenously, EPO can result in hypertension, thrombosis, and stroke [16]. To have activity in the brain, EPO must be intravenously administered at high levels, which can result in edema and cerebral hemorrhage [17]. Therefore, in order to avoid these adverse events new strategies are needed to precisely deliver EPO to the brain.

NeuroEPO plus (NeuralCIM^®^, Center of Molecular Immunology Havana Cuba) is a recombinant sialo-glycoprotein with low sialic acid content. NeuroEPO plus showed an increase in bi- and triantennary structures to detriment of tetraantennary with additional LacNAc units. This characteristic glycosylation detected in neuroEPO plus could explain the higher efficiency of this non-erythropoietic erythropoietin in the mechanisms of neuroprotection and neuroregeneration described in vitro and in vivo [18].

The reduction in sialic acid causes rapid hepatic degradation, and thus neuroEPO plus needs to be administered by the intranasal route to avoid hepatic first pass metabolism and degradation in the gastrointestinal tract [19]. NeuroEPO plus is a neuroprotective agent that activates multiple signaling pathways to inhibit apoptosis, reduce cellular neuronal death, inflammation, and local edema. NeuroEPO plus induces neuroglobin protein synthesis selectively in the damaged regions and increases angiogenesis and extension of capillaries, which protects the vascular endothelium. NeuroEPO plus activity contributes to neurogenesis and neuroplasticity that controls homeostasis and rescues brain functions damaged by brain injury [20–22].

In the ICV-Aβ25-35 AD mice model and in the aged transgenic mice model Tg2576, neuroEPO plus prevented neuronal loss in the hippocampal CA1 region, reduced the number of brain amyloid plaques, reduced the increment of TNF-α, IL-1β levels, glial activation, neuroinflammation, oxidative effects, and apoptosis in neurons and, also, significantly improved spatial memory [23, 24].

Direct evidence of neuroEPO plus in the CNS has been demonstrated in *Mongolian gerbils* using radiolabeled neuroEPO plus [25, 26]. Indirect evidence of neuroEPO plus in the cerebral spinal fluid (CSF) is supported by studies measuring total EPO in the CSF of non-human primates [26] and in patients with Spinocerebellar ataxia type 2 (SCA2) [27]. These studies suggest that neuroEPO plus is reaching the intended target when delivered intranasally.

NeuroEPO plus has been safely evaluated in healthy volunteers [28], patients with SCA2 [27] and patients with Parkinson disease [29]. We conducted a phase 2–3 trial (ATHENEA), to assess the safety and efficacy of neuroEPO plus in participants with mild-to-moderate Alzheimer’s clinical syndrome.

## Material and methods

### Trial conduct

ATHENEA was a 48-week, phase 2–3, randomized, double-blind, parallel, multicenter, adaptive, placebo-controlled trial with participants screened in Havana Cuba from September, 2017 to September, 2020. The responsible investigator or the site coordinator in each clinical site enrolled the participants.

All patients had the opportunity to drop-out of research. The sponsor CIM designed the trial and analyzed the data in collaboration with the academic authors, provided neuroEPO plus and placebo and aided in drafting the manuscript. Confidentiality agreements were in place between the sponsor and the authors and site investigators. CIM provided partial funding for the trial.

An independent data and safety monitoring board, consisting of experts in Alzheimer’s disease, clinical trial, and statistics, provided trial oversight. Clinical assessment raters were unaware of the trial-group assignments. All the authors vouch for the completeness and accuracy of the data, the fidelity of the trial to the protocol (available at https://rpcec.sld.cu/trials/RPCEC00000232-En).

Data were blindly gathered by the study investigators, blindly analyzed by the sponsor, and interpreted by the sponsors in collaboration with the researchers once the randomization was opened.

### Trial design and participants

The trial included participants age ≥ 50 years with Alzheimer’s clinical syndrome (—recommended terminology for clinically ascertained multi- (or single-) domain amnestic syndrome or a classic syndromal variant (what has historically been labeled “possible or probable AD”)) [30], on the basis of National Institute on Aging—Alzheimer’s Association (NIA-AA) 2011criteria, the diagnosis was performed considering only clinical criteria [31].

Eligible participants had a Global Deterioration Scale (GDS) score of 3 to 5 (inclusive). Procedures also included magnetic resonance imaging (MRI) consistent with AD [32–35]. Key exclusion criteria included neurologic disease other than AD and presence of imaging abnormalities (brain tumor, head trauma, any intracerebral hemorrhage greater than 1 cm^3^, two or more lacunar infarcts, more than 1 area of superficial siderosis) on MRI.

In addition, cell DNA was extracted and apolipoprotein E (APOE) genotype determined by PCR, following the standard protocol for determination of the APOE ε4 carrier or non-carrier.

### Randomization and intervention

**Fig. 1:**
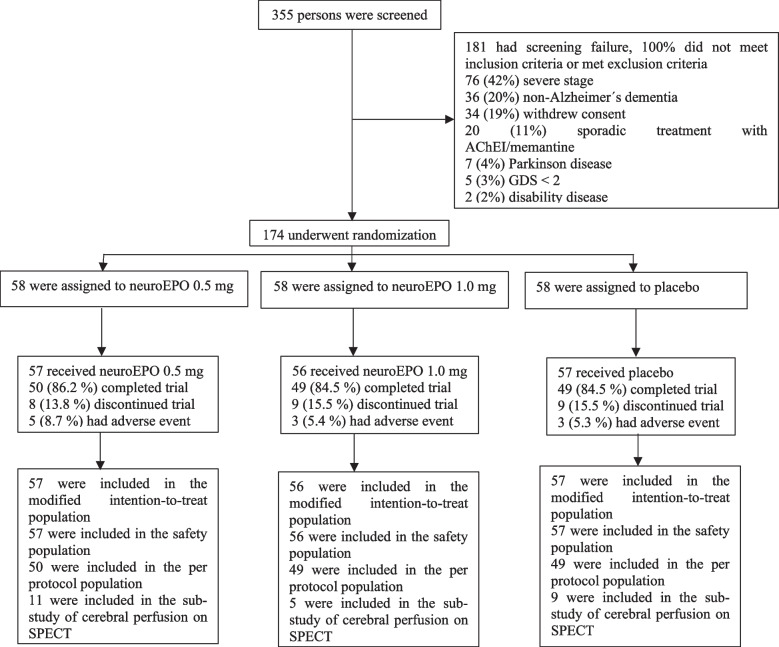
Screening, randomization, and follow-up. Participants who completed at 48 weeks are considered to have completed the trial (per protocol population). The modified intention-to-treat population which included participants with at least one dose of neuroEPO plus or placebo and a baseline measurement based on randomized treatment. The per protocol population which included subjects who complied with the protocol sufficiently (more than 90% of treatment with efficacy outcomes at baseline and at 48 weeks without any major deviation of protocol) to ensure that these data would be likely to exhibit the effects of treatment according to the underlying scientific model. Subjects were considerate in their randomized group. The safety population included participants who received at least one dose of neuroEPO or placebo. SPECT denotes single-photon emission computed tomography

Eligible participants were randomly assigned in a 1:1:1 ratio (Fig. 1) by a computer-generated sequence using R v.3.2.4 (RCore Team, 2016), with adaptive randomization by ADAS-Cog11 and GDS, to minimize the imbalance between the groups. A first randomization block size was 6. From this point, a case-by-case randomization was made based on the two covariates mentioned above. The head of the pharmacy department at the sponsor site assigned participants to interventions.

Randomized participants received either neuroEPO plus 0.5 mg or neuroEPO plus 1.0 mg or placebo 3 times a week (Monday, Wednesday, and Friday), during 4 weeks. In order to keep the blinding, placebo was divided, and a half received 0.5 mL and the other 1.0 mL. From the 5 to 48 weeks, neuroEPO plus groups received 0.5 mg and placebo group received 0.5 mL.

### Outcomes

The primary outcome was change in the score on the 11-item cognitive subscale of the Alzheimer’s Disease Assessment Scale (ADAS-Cog11) from baseline to 48 weeks. The ADAS-Cog11 score is a validated outcome measure used in clinical trials of Alzheimer’s. It includes 11 tasks that include both subject-completed tests and observer-based assessments. Together these tasks assess the cognitive domains of memory, language, and praxis [36, 37]. Total scores range from 0 to 70, higher scores indicating greater impairment and with a score of 16 to 45 indicating mild-moderate stage.

Secondary outcomes included changes from baseline to 48 weeks by the Clinician Interview-Based Impression of Change Incorporating Caregiver Information (CIBIC+; range, 0 to 7, with higher scores indicating greater impairment) [38], the GDS (range, 1 to 7, with higher scores indicating greater impairment) [39], Montreal Cognitive Assessment (MoCA; normal ≥ 26/30) [40], Neuropsychiatric Inventory (NPI; range, 0 to 120, higher scores reflect greater severity) [41] and Activities of Daily Living Scales (ADL: Katz, Barthel and Lawton; with lower scores indicating greater impairment) [42].

The changes from baseline to 48 weeks in cerebral perfusion by single-photon emission computed tomography (SPECT), lower cerebral perfusion in the posterior emporoparietal regions which is relevant to the physiology of AD [43]; volumetric MRI (vMRI) rates of hippocampal atrophy which are sensitive markers of neurodegeneration in AD [32–35], and adverse events were additional secondary outcomes.

The clinical outcomes were measured at baseline, 24 and 48 weeks. Brain perfusion and hippocampal volume were measured at baseline and 48 weeks.

### SPECT and MRI acquisition

The SPECT and MRI acquisition and analysis are included in Methods in Supplement.

### Sample size calculation

The sample size for this trial was estimated on the basis of comparison of neuroEPO plus and placebo with respect to the primary efficacy outcome, the change from baseline at 48 weeks in the ADAS-Cog11 score. A reduction of at least 3 points between the changes of the ADAS-Cog11 total score in the neuroEPO plus vs. the placebo group was hypothesized. Therefore, after assuming 20% discontinuation rate, 80% power to achieve statistical significance at a 2-sided *α* level of 0.025, the total planned enrolled was 114, including 38 participants in each group neuroEPO plus (0.5 or 1.0 mg) and placebo.

An interim analysis for futility or efficacy was planned, when the first 20 participants of each group were included and evaluated at 24 weeks. At that moment, a blinded readjustment of the sample size based on the observed variance was considered. This adjustment was necessary and the sample size was increased by 60 (20 patients by group). The final sample size was 174 (58 patients by group).

Additional information is provided in Methods in Supplement.

### Statistical analysis

All analyses were performed using the software programs IBM SPSS Statistics for Windows, version 25.0 (Armonk, NY: IBM Corp) and R version 3.2.4.

#### Efficacy analyses

The principal outcome was defined by ADAS-Cog11.

Efficacy analyses (ADAS-Cog11 and CIBIC+) were performed in the modified intention-to-treat population (mITT, participants with at least one dose of neuroEPO plus or placebo and a baseline measurement based on randomized treatment).

Secondary efficacy analyses (ADAS-Cog11, CIBIC+, GDS, MoCA, NPI, ADL, hippocampal volume, and brain perfusion) were performed in the per protocol population (PP, subjects who complied with the protocol sufficiently to ensure that these data would be likely to exhibit the effects of treatment according to the underlying scientific model).

Goodness-of-fit to the normal distribution was evaluated using the Kolmogorov-Smirnov test. The variables used did not meet the above assumption. Therefore, they are summarized using the median, as well as the 95% confidence intervals for the differences with respect to the placebo group. Comparisons were made using Kruskal-Wallis test and Dunn test for multiple comparisons. With the qualitative variables, the dependency relationship regarding the treatment was studied using the chi-square test.

It was planned that if the probability of the fulfilled the hypothesis at 24 weeks was small *P*_*(Ɵ*>*2)*_ < *0.1* (*Ɵ* is the final distribution of the difference in the ADAS-Cog11 score neuroEPO plus – placebo), the evaluation of the hypothesis will be postponed to 48 weeks. After the interim analysis, the previous criteria were satisfied and the hypothesis was evaluated at 48 weeks.

#### Sensitivity analyses

Considering that the condition of the patients deteriorates over time, as a sensitivity analysis, two imputation methods for the missing data were proposed for the primary variable (ADAS-Cog11) and secondary variable (CIBIC+). The methods were as follows (1) regression analysis and (2) analysis with the worst response (missing values were considered as “non-responder” with an increase in the value of the initial score by 5 and 10 units for ADAS-Cog11 at 24 and 48 weeks, respectively, and 7 units for CIBIC+).

#### Adverse events

Safety was evaluated in the safety population, participants who received at least one dose of neuroEPO plus or placebo. Safety evaluations included monitoring of adverse events, vital signs, physical examinations and clinical laboratory variables. Adverse events were collected from the first administration of study drug until the patient’s final visit in the study and were summarized according to event frequency by treatment assignment. Frequency distributions by treatments were estimated. No safety monitoring of the MRI data was done.

## Results

### Trial population and baseline characteristics

A total of 355 participants were screened at two sites in Cuba from September 2017 through August 2019. In total, 174 patients (mean age, 74.0 years; 121 [71.2%] women) were enrolled and 85% completed the trial: 50 (86.2%) in the neuroEPO plus 0.5 mg, 49 (84.5%) in the neuroEPO plus 1.0 mg, and 49 (84.5%) in the placebo group (Fig. 1).

The majority of patients screened, but not enrolled, were the result of severe stage, non-Alzheimer dementia, no informed consent, and sporadic treatment with acetylcholinesterase inhibitors (AChEIs)/memantine. The most common primary reasons for treatment withdrawal were voluntary discontinuation (10 patients, 5.74%) and protocol non-adherence (9 patients, 5.17%) (Fig. 1). Baseline characteristics are summarized by treatment groups (*n* = 170) (Table 1). These characteristics were similar to what has been observed in population studies involving persons with Alzheimer’s disease.

**Table 1:** Baseline demographics and clinical characteristics in modified intention-to-treat population

**Characteristic**	**NeuroEPO plus** **0.5 mg (*n* = 57)**	**NeuroEPO plus** **1.0 mg (*n* = 56)**	**Placebo (*n* = 57)**	**Total (*n* = 170)**
Age median (IR) — yr	75.0 ± 10.0	73.0 ± 10.0	74.0 ± 14.0	74.0 ± 9.0
Sex — no. (%)				
Female	38 (66.7)	41 (73.2)	42 (73.7)	121 (71.2)
Male	19 (33.3)	15 (26.8)	15 (26.3)	49 (28.8)
Race — no. (%)^a^				
White	43 (75.4)	49 (87.5)	53 (93.0)	145 (85.3)
Black	6 (10.5)	2 (3.6)	0	8 (4.7)
Mestizo	8 (14.0)	5 (8.9)	4 (7.0)	17 (10.0)
Educational level — no. (%)				
None	12 (21.1)	9 (16.1)	12 (21.1)	33 (19.4)
Elementary	17 (29.8)	20 (35.7)	15 (26.3)	52 (30.6)
Junior	9 (15.8)	13 (23.2)	16 (28.1)	38 (22.4)
Senior	8 (14.0)	13 (23.2)	8 (14.0)	29 (17.1)
University	11 (19.3)	1 (1.8)	6 (10.5)	18 (10.6)
Time since diagnosis median (IR)— yr	1.0 ± 2.0	1.0 ± 2.0	2.0 ± 1.0	1.0 ± 2.0
ADAS-Cog11 score^b^ median ± IR	22.0 ± 12.0	22.5 ± 14.0	23.0 ± 13.0	22.0 ± 12.0
Range	10 to 53	10 to 46	11 to 47	10 to 53
GDS score — no. (%)^c^				
2	0	1 (1.8)	1 (1.8)	2 (1.2)
3	31 (54.4)	25 (44.6)	26 (45.6)	82 (48.2)
4	25 (43.9)	26 (46.4)	27 (47.4)	78 (45.9)
5	1 (1.8)	4 (7.1)	3 (5.3)	8 (4.7)
Stage — no. (%)				
Mild	37 (64.9)	32 (57.1)	36 (63.2)	105 (61.8)
Moderate	20 (35.1)	24 (42.9)	21 (36.8)	65 (38.2)
APOE ε status — no. (%)				
No. of participants evaluated	**(*n* = 29)**	**(*n* = 30)**	**(*n* = 34)**	**(*n* = 93)**
Carrier	16 (55.2)	16 (53.3)	12 (35.3)	44 (47.3)
Non-carrier	13 (44.8)	14 (46.7)	22 (64.7)	49 (52.7)

The analysis was performed in the modified intention-to-treat population, which included participants with at least one dose of neuroEPO plus or placebo and a baseline measurement based on randomized treatment

*ApoE* Apolipoprotein E, *yr* Years, *No.* Number, *IR* Interquartile range

^a^Race was determined by the participants

^b^Scores on the 11-item cognitive subscale of the Alzheimer’s Disease Assessment Scale (ADAS-Cog11) range from 0 to 70, with higher scores indicating greater impairment (scores were adjusted for age and formal education)

^c^Scores on the Global Deterioration Scale (GDS) range from 1 to 7, with higher scores indicating greater impairment

As seen in the Table 1, the groups are balanced and are similar in their characteristics. The percentage of APOE4 non-carrier subjects in the control group, compared to those treated, may draw attention. However, the apparent imbalance observed is not significant (*P* = 0.206).

### Primary outcome

**Fig. 2:**
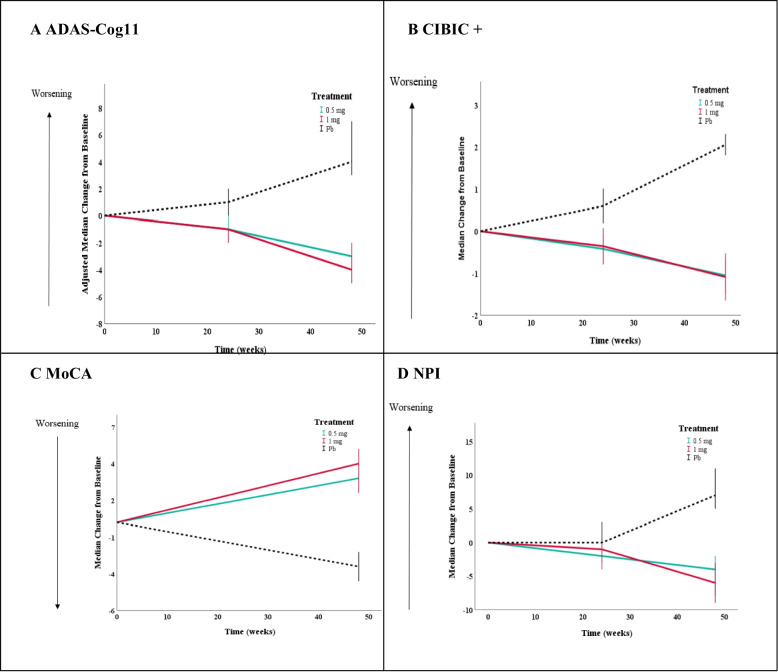
Primary and secondary outcomes from baseline to 48 weeks. Panels **A** and **B** show results in the modified intention-to-treat population (0.5 mg: *n* = 57; 1 mg: *n* = 56; Pb: *n* = 57). Panels **C** and **D** show results in the per protocol population (0.5 mg: *n* = 50; 1 mg: *n* = 49; Pb: *n* = 49). Panel **A** shows the results for the primary outcome, the score on the 11-item cognitive subscale of the Alzheimer’s Disease Assessment Scale (ADAS-Cog11; range, 0 to 70, with higher scores indicating greater impairment). Panels **B**, **C**, and **D** show the results for the secondary outcomes. Panel **B** shows results for the change from baseline in the score on the Clinician Interview-Based Impression of Change Incorporating Caregiver Information (CIBIC+; range 0 to 7, with higher scores indicating greater impairment). Panel **C** shows results for the change from baseline in the score on the Montreal Cognitive Assessment (MoCA; normal ≥ 26/30, with lower scores indicating greater impairment). Panel **D** shows results for the change from baseline in the score on the Neuropsychiatric Inventory (NPI; range 0 to 120, higher scores reflect greater severity). 95% CIs for median changes were calculated (data was not approximated by normal distribution)

The adjusted median change from baseline in the ADAS-Cog11 score at 48 weeks was −3.0 (95% CI, −4.3 to −1.7) in the 0.5 mg neuroEPO plus group, −4.0 (95% CI, −5.9 to −2.1) in the 1.0 mg neuroEPO plus group, and 4.0 (95% CI, 1.9 to 6.1) in the placebo group. Difference vs. placebo was 7.0 points (95% CI, 4.5 to 9.5) in the 0.5 mg neuroEPO plus group, *P* = 0.000, and 8.0 points (95% CI, 5.2 to 10.8) in the neuroEPO plus 1.0 mg, *P* = 0.000 (Fig. 2A and Table 2).

**Table 2:** Clinical outcomes from baseline to 48 weeks

**Outcomes**	**NeuroEPO plus** **0.5 mg**	**NeuroEPO plus** **1.0 mg **	**Placebo**
**Primary efficacy outcome**			
Change from baseline to 48 weeks in the ADAS-Cog11 score			
No. of participants evaluated^a^	57	56	57
Adjusted median change	−3.0	−4.0	4.0
Adjusted absolute median difference vs. placebo (95% CI)	7.0 (4.5 to 9.5)	8.0 (5.2 to 10.8)	
*P* value vs. placebo	0.000	0.000	
**Secondary efficacy outcomes**			
Change from baseline to 48 weeks in the CIBIC+ score			
No. of participants evaluated^a^	57	56	57
Median change	−1.0	−1.0	2.0
Absolute median difference vs. placebo (95% CI)	3.0 (2.2 to 3.7)	3.0 (2.0 to 3.9)	
*P* value vs. placebo	0.000	0.000	
Change — no. (%)			
Improvement	40 (70.2)	39 (69.6)	0
No change	13 (22.8)	8 (14.3)	1 (1.8)
Worsening	4 (7.0)	9 (16.1)	56 (98.2)
Change from baseline to 48 weeks in the GDS score			
No. of participants evaluated^b^	50	49	49
Change — no. (%)			
No increase	45 (90.0)	47 (95.9)	34 (69.4)
Increase	5 (10.0)	2 (4.1)	15 (30.6)
Difference vs. placebo (95% CI)	19.9 (4.9 to 34.8)	25.7 (11.6 to 40.3)	
*P* value vs. placebo	< 0.005	< 0.005	
Change from baseline to 48 weeks in the MoCA			
No. of participants evaluated^b^	50	49	49
Median change	3.0	4.0	−3.0
Median difference vs. placebo (95% CI)	6.0 (4.7 to 7.3)	7.0 (5.4 to 8.6)	
*P* value vs. placebo	< 0.005	< 0.005	
Change from baseline to 48 weeks in the NPI			
No. of participants evaluated^b^	50	49	49
Median change	−4.0	−6.0	7.0
Absolute median difference vs. placebo (95% CI)	11.0 (6.9 to 15.1)	13.0 (8.3 to 17.7)	
*P* value vs. placebo	< 0.005	< 0.005	
Change from baseline to 48 weeks in the perfusion on the temporoparietal region			
No. of participants evaluated^b^	11	5	9
Change — no. (%)			
Improvement	7 (63.6)	2 (40.0)	0 (0)
No change	3 (27.3)	2 (40.0)	7 (77.8)
Worsening	1 (9.1)	1 (20.0)	2 (22.2)

Scores on the 11-item cognitive subscale of the Alzheimer’s Disease Assessment Scale (ADAS-Cog11) range from 0 to 70, with higher scores indicating greater impairment (scores were adjusted for age and formal education)*.* Scores on the Clinician’s Interview Based Impression of Change Incorporating Caregiver Information (CIBIC+) range from 0 to 7, with higher scores indicating greater impairment. Scores on the Global Deterioration Scale (GDS) range from 1 to 7, with higher scores indicating greater impairment. Scores on the Montreal Cognitive Assessment (MoCA) ≥ 26/30 normal. Scores on the Neuropsychiatric inventory (NPI) range from 0 to 120, higher scores reflect greater severity

*No.* Number, *IR* Interquartile range, *CI* Confidence interval

^a^The analysis was performed in the modified intention-to-treat population (worst scenario, increase of 10 and 7 points at week 48 in ADAS-Cog11 and CIBIC+ respectively), which included participants who received at least one dose of neuroEPO or placebo and who had a baseline assessment

^b^The analysis was performed in the per protocol population, which included subjects who complied with the protocol sufficiently (more than 90% of treatment with efficacy outcomes at baseline and at 48 weeks without any major deviation of protocol) to ensure that these data would be likely to exhibit the effects of treatment according to the underlying scientific model. Subjects were considerate in their randomized group

The clinical significance was evaluated considering 4-point change in ADAS-Cog score. The 49.1 and 58.9% of neuroEPO plus 0.5 mg and 1.0 mg, respectively decreased ADAS-Cog11 values in ≥ 4 units (difference, 47.4 (95% CI, 34.0 to 60.8) in neuroEPO plus 0.5 mg; *P* = 0.000 and 57.2 (95% CI, 43.8 to 70.5) in neuroEPO plus 1.0 mg; *P* = 0.000). The 86% of placebo-treated patients increased ADAS-Cog11 values in ≥ 2 units (Fig. S1, Supplement).

Sensitivity analyses showed similar results in PP population and mITT using regression models and the worst response (Fig. S2, Supplement).

### Secondary outcomes

#### Clinical assessments

The differences between treatment groups and placebo in the median change from baseline at 48 weeks were 3.0 (95%CI, 2.2 to 3.7) in neuroEPO plus 0.5 mg, *P* < 0.001, and 3.0 (95%CI, 2.0 to 3.9) in neuroEPO plus 1.0 mg, *P* < 0.001, for CIBIC+; 6.0 (95%CI, 4.7 to 7.3), *P* < 0.005, in neuroEPO plus 0.5 mg and 7.0 (95%CI, 5.4 to 8.6) in neuroEPO plus 1.0 mg, *P* < 0.005, for MoCA; and 11.0 (95%CI, 6.9 to 15.1), *P* = 0.005, in neuroEPO plus 0.5 mg and 13.0 (95%CI, 8.3 to 17.7) in neuroEPO plus 1.0 mg, *P* = 0.005, for NPI (Fig. 2B–D and Table 2). About GDS, the differences between treatment groups and placebo in the percentage change from baseline to 48 weeks were 19.9 (95%CI, 4.9 to 34.8) in neuroEPO plus 0.5 mg, *P* = 0.005, and 25.7 (95%CI, 11.6 to 40.3) in neuroEPO plus 1.0 mg, *P* = 0.005 (Table 2).

With respect to ADL, there was not an important variation at week 48. Most patients did not change their initial functional status. However, in the Lawton scale, neuroEPO plus-treated patients maintained the same median value (6.0 ± 4.0), whereas patients in the placebo group decreased the median value by 1 point (5.0 ± 3.0), Table S1, Supplement.

#### Cerebral perfusion

The sub-study of cerebral perfusion involved only 28 participants, 25 (89.3%) completed the trial: 11 (44%) neuroEPO plus 0.5 mg, 5 (20%) neuroEPO plus 1.0 mg, and 9 (36%) placebo. At baseline, all subjects showed evidence of low cerebral perfusion. At 48 weeks, 63.6% of neuroEPO plus 0.5 mg, 40% of neuroEPO plus 1.0 mg, and 22.2% of placebo group improved their global cerebral perfusion. These results were not significant, *P* = 0.345, possibly due to the small sample size (Table S2, Supplement). However, in the temporoparietal region, none of the placebo group improved whereas 9 subjects of neuroEPO plus groups had an improvement of their cerebral perfusion, *P* < 0.016) (Table 2). The Figs. S3-1, S3-2 and S3-3, Supplement show SPECT sequences of 6 subjects before and after treatment.

#### Volumetric MRI

Ninety-eight subjects completed the study: 35 (35.7%) neuroEPO plus 0.5 mg, 31 (31.6%) neuroEPO plus 1.0 mg, and 32 (32.7%) placebo. At baseline, the hippocampal (HC) volume measured by MRI was adjusted by estimating the total intracranial volume (eTIV) and it was 2.06 × 10^−3^ (95% IC 1.99 to 2.23) for the left HC and 2.10 × 10^−3^ (95% IC 2.02 to 2.17) for the right HC. At 48 weeks, the HC volume was 1.99 (95% IC 1.91 to 2.06) in the case of the left HC and 2.04 × 10^−3^ (95% IC 1.96 to 2.12) for the right HC. The HC volume by group is presented in the Table S3, Supplement.

For the three groups at 48 weeks, vMRI showed a decrease in the hippocampal volume. The three groups had a similar global percentage of change: −3.40, −3.26, and −3.32 in the case of neuroEPO plus 0.5 mg, 1.0 mg, and placebo, respectively, *P* < 0.98 (Table S4, Supplement). There was no difference seen between groups, possibly given the cohort size.

The analysis of HC volume perceptual change vs. ADAS-Cog11 initial value for individual subjects (Fig. S4, Supplement) showed a correlation between the progression of HC atrophy and the initial cognitive status of the patient, only in the placebo group (*P* = 0.0053). The magnitude of the percent change at 48 weeks was similar in the treated groups without significant differences (*P* = 0.61 and *P* = 0.19 to neuroEPO plus 0.5 and 1.0 mg, respectively).

#### Adverse events

Eleven patients (6.5%) had adverse events: 5 (8.8%), 3 (5.4%), and 3 (5.3%) in the neuroEPO plus 0.5 mg, 1.0 mg, and placebo group, respectively.

The incidence of death was 1.8% in the neuroEPO plus 0.5 mg group and 1.8% in the placebo group. No deaths were considered by the investigators to be related to neuroEPO plus. Numbness in the upper right member was the only adverse events related with neuroEPO plus 0.5 mg (Table 3).

**Table 3:** Summary of adverse events (AEs) by treatment group

**Event**	**NeuroEPO plus** **0.5 mg (*n* = 57)**	**NeuroEPO plus** **1.0 mg (*n* = 56)**	**Placebo (*n* = 57)**
**Overview of AE — no. (%)**			
Participants with any AE	5 (8.8)	3 (5.4)	3 (5.3)
AE related to neuroEPO plus or placebo	1 (1.8)	0	0
Serious AE	3 (5.3)	0	3 (5.3)
Death (not related to treatment)	1 (1.8)	0	1(1.8)
Participants with ≥ 1 serious AE	1 (1.8)	0	1(1.8)
**AE that occurred in either group**			
Bronchopneumonia	1(1.8)	0	1(1.8)
Pulmonary embolism	1 (1.8)	0	0
Numbness upper right member	1 (1.8)	0	0
Bronchopneumonia	1 (1.8)	0	0
Fall down	0	0	1(1.8)
Headache	1 (1.8)	0	0
Nasal congestion	1 (1.8)	0	0
Constipation	1 (1.8)	0	0
Dehydration	0	0	1(1.8)
Pain due to fall down	0	0	1(1.8)
Flu status	0	1 (1.8)	0
Hypertension	0	1 (1.8)	0
Hematoma due to fall down	0	0	1 (1.8)
Urinary sepsis	0	1 (1.8)	0
Irritability	0	0	1 (1.8)
Palpitations	1 (1.8)	0	0
Deep venous thrombosis	1 (1.8)	0	0
Vomiting	1 (1.8)	1 (1.8)	1 (1.8)
Diarrhea	0	1 (1.8)	0

The analysis was performed in the safety population, which included participants who received at least one dose of neuroEPO or placebo

*AEs* Adverse events, *No.* Number

The incidence of serious adverse events was 5.3% in the neuroEPO plus 0.5 mg group and in the placebo group. In the neuroEPO plus 0.5 mg group, 1 participant with bronchopneumonia, deep venous thrombosis, and pulmonary embolism subsequently died. In the placebo group, 1 participant with vomiting, dehydration, and bronchopneumonia subsequently died.

The most commonly reported serious adverse events were bronchopneumonia (1.8% in the neuroEPO plus 0.5 mg and in the placebo group), pulmonary embolism (1.8% in the neuroEPO plus 0.5 mg group), deep venous thrombosis (1.8% in the neuroEPO plus 0.5 mg group), vomiting (1.8% in the placebo group), and dehydration (1.8% in the placebo group).

There were 21 adverse events, 10 in neuroEPO plus 0.5 mg, 5 in neuroEPO plus 1.0 mg, and 7 in placebo group. The most common adverse events were vomiting (1.8% each in neuroEPO plus 0.5 mg, 1.0 mg, and in placebo) and bronchopneumonia (1.8% in neuroEPO plus 0.5 mg and in placebo group). The overall incidence of adverse events was similar in the three groups (Table 3).

Treatment discontinuation due to adverse events was reported just in the two deceased subjects.

NeuroEPO plus produced no clinically relevant changes in laboratory tests, specifically no clinically important hemoglobin variation was detected and no difference between groups were observed at 48 weeks (Table S5 and Fig. S5, Supplement). Besides, no clinically relevant changes on physical examination or in vital signs were observed.

The benefit-risk analysis showed striking evidence in favour of the benefit. The odds were greater than 400 for neuroEPO plus groups indicating that the probability of benefit is greater than the probability of risk; for placebo, the odds was greater than 70 (Fig. S6, Supplement).

## Discussion

In this phase 2–3 trial, neuroEPO plus groups slowed the Alzheimer’s clinical syndrome progression, based on the ADAS-Cog11 score, compared with placebo and across secondary clinical outcomes including CIBIC+, GDS, MoCA, NPI, and cerebral perfusion.

NeuroEPO plus treatment resulted in a clinically meaningful benefit (considered as the minimum of a clinically important effect of 4 points in the primary end point of the ADAS-Cog11 score) [44]. This trial used a definition of meaningful within patient change (MWPC) [45] based on the proposal of the panel of experts from the FDA: minimum of a clinically important effect of 4 points in the ADAS-Cog score [44]. In this trial, considering the ITT population worst scenario, 54% of the neuroEPO plus-treated patients decreased the ADAS-Cog11 scores in more than 4 units, suggesting some levels of restoration of the cognitive function. On the other hand, 71.9 and 68.1% of the subjects from neuroEPO plus 0.5 and 1.0 mg, respectively, stabilized the ADAS-Cog11 scores (Fig. S1, Supplement). This is an important finding, because to control the disease and delay progression are the primary objectives of current AD drug development pipeline.

Additionally, 69.9% of the patients receiving neuroEPO plus improved the CIBIC+ while 98.2% of the placebo patients worsened the CIBIC+ score. Furthermore, an estimated 92.9% of participants receiving neuroEPO plus groups had no change in the GDS at 48 weeks (no disease progression), compared with 69.4% of participants receiving placebo.

In our trial, the adjusted absolute difference in the changes between the neuroEPO plus groups and placebo group goes far beyond the initial hypothesis.

The potential benefit of anti-Aβ drugs to cognition in AD remains under active debate [46, 47], despite the fact that discrete benefits have been observed in clinical trials developed with anti-amyloid drugs [3, 4, 6–8], that is encouraging and justify the conduct of new clinical trials.

Perhaps this discrete benefit is due to the fact that these drugs have been directed at a single target. Something similar happens with other previously registered medications [48]. In our case, non-clinical studies have shown that neuroEPO plus has an effect on different therapeutic targets and this could explain our results [23–26].

NeuroEPO plus treatment shows good results for both moderate and mild stage (Table S6 and Fig. S7, Supplement). This is unprecedented, because to achieve some control of moderate-stage disease, with the currently available drugs, is very difficult [49]. Besides, disappointing clinical trials over the last several years have led to a growing consensus on the need to intervene earlier in the disease process, prior to the onset of any clinical symptoms. In this sense, our results showed better effect in mild patients, suggesting that an earlier use of neuroEPO plus could be more beneficial. However, the clinical effect in the moderate stage is important and perhaps could be further consolidated with a longer period of neuroEPO plus treatment or its combination with some other therapies.

In our patient set, the frequency of subject with APOE ε4 allele positive was lower than the reported in literature for patients with a definitive diagnostic of AD (47% vs. greater than 60%) [8, 46] and the most frequent APOE genotype was ε3/ε3 with 44 subjects (47.3%). These results are similar to other studies carried out in the Cuban population [50–55]. On the other hand, emerging research has shown racial and ethnic variations in the magnitude of association between the APOE ε4 allele and the risk of developing AD [50]. In our trial, the majority of subjects (allele 4 carriers and non-carriers) treated with neuroEPO plus responded to the treatment, whereas the majority of subjects (APOE4 carriers and non-carriers) in the placebo group worsened. Therefore, being a carrier or not of allele 4 did not influence in the results of the study. There was no dependence between the response to treatment and the APOE genotype, even though the percentage of genotyped individuals is relatively small (Table S7, Supplement).

Cerebral perfusion measured by SPECT is a non-invasive image diagnosis method used to evaluate functional parameters of brain and is typically reduced in Alzheimer patients. Flow reduction in the posterior temporoparietal regions is particularly relevant [43]. After 48 weeks of treatment, 9 (56.3%) participants from the neuroEPO plus groups improved their perfusion in all cerebral lobules, including the temporoparietal region, and 2 (22.2%) participants from the placebo group improved their cerebral perfusion just in the frontal lobe [56].

At baseline, the HC volume measured by MRI was significantly lower (*P* = 0.000) than Cam-CAN database for healthy individuals [57]. This result correlated with the pattern observed in AD subjects [32–35].

Changes in vMRI showed that the HC volume was independent of the ADAS-Cog11 score at baseline. However, the finding that the magnitude of the percent change was significant at 48 weeks in the placebo group is important, and additional trials with a larger sample size and over a longer period of time are needed to evaluate this aspect.

NeuroEPO plus treatment shows a very good safety profile, especially when compared with other drugs approved for the treatment of AD, where a greater number of adverse events, many of them serious, have been reported [48, 58–60].

Oral administration of AChEIs (donepezil, galantamine, and rivastigmine) increases gastrointestinal adverse effects, such as abdominal pain, nausea, vomiting, diarrhea, and poor appetite. Also, older adults treated with AChEIs are at greater risk of cardiovascular side effects such as sinoatrial and atrioventricular block, severe sinus bradycardia, and QT interval prolongation with torsades de pointes [61].

Some gastrointestinal and nervous system side effects such as nausea, vomiting, diarrhea, anorexia, dizziness, depression, and headache were observed with the use of memantine [48].

Anti-Aβ drugs cause MRI-detectable ARIA. These side effects are often clinically silent or are associated with non-life threating symptoms such as migraine that resolve over 3–4 months of treatment suspension. In severe cases, ARIA may require hospitalization and some patients interrupt treatment. A meta-analysis revealed the potential for anti-Aβ therapies to compromise long-term brain health by accelerating brain atrophy [47, 59–61].

In our trial, the majority of adverse events reported, were mild and no related with neuroEPO plus treatment. The drug was well tolerated and no serious related events were reported. NeuroEPO plus is a derivative of EPO, which is known to cause hematological toxicity. During trial, the behavior of the hematological parameters was carefully evaluated. Hemoglobin slightly decreased over time in the three groups, but within the normal values, with no statistically significant differences between the groups or over time. Therefore, intranasal administration of neuroEPO plus did not cause any sign of hematological toxicity. These safety results are consistent with previous clinical trials [27–29].

### Limitations

This study has some limitations. The sample size was relatively small. The data collection was done for 48 weeks. The cerebral perfusion was evaluated in a reduced number of patients. The trial was conducted during the COVID-19 pandemic and encountered obstacles including missed doses, delayed assessments, and intercurrent illnesses. There are no pharmacokinetic studies in AD subjects. However, evidence for the presence of neuroEPO plus in the CSF has been observed when neuroEPO plus was administered in subjects with SCA2 [27]. In non-clinical studies, it was observed that only 0.026% is absorbed. Therefore, extrapolating to humans, the subject would be receiving 20 and 40 mU/mL for 0.5 and 1.0 mg, respectively [26]. Pharmacokinetic study will be performed in AD subjects, once radiolabeled neuroEPO plus is achieved to differentiate from endogenous EPO, which is very difficult. The trial did not include molecular biomarkers for the definitive diagnosis of AD. The clinical trial began in 2017. The trial used the NIA-AA 2011 diagnostic criteria, where the use of biomarkers was not mandatory [31]. In 2018, the NIA-AA and FDA recommended the use of biomarkers for the diagnosis of AD in the framework of research. Still, they acknowledged that, in current medical practice, the diagnosis is frequently only clinical since access to biomarkers is not easy and their use is expensive [30].

Additional trials of neuroEPO plus include a 78-week phase 3 comparing neuroEPO plus vs. donepezil or the combination, where the clinical diagnosis will be complemented with the determination of β-amyloid in CSF (RPCEC number, RPCE00000409) and a 104-week phase 3 long-term extension trial (RPCEC00000410) in mild-to-moderate Alzheimer’s disease patients.

## Conclusions

In patients with mild-to-moderate Alzheimer’s clinical syndrome, neuroEPO plus reduced ADAS-Cog11 score, without related serious adverse events. NeuroEPO plus was approved using the accelerated approval pathway, by the Cuban Regulatory Authority, sanitary register number B-22-016-N07-C. Longer trials are warranted to determine the efficacy and safety of neuroEPO plus in early Alzheimer’s disease.

### Supplementary Information

**Additional file 1:** Supplement Methods and Results. **Table S1.** Secondary outcomes (ADL) from Baseline to 48 Weeks. **Table S2.** Changes in global cerebral perfusion from baseline to week 48. **Table S3.** Initial and final hippocampal volume adjusted by eTIV. **Table S4.** Percentage of change in the hippocampal volume adjusted by eTIV. **Table S5.** Hemoglobin variation, Bonferroni-corrected (adjusted alpha error level of 0.017). **Table S6.** Primary outcome ADAS-Cog11 score by stage. **Table S7.** Clinical response (ADAS-Cog value) stratified by APOE genotype and groups. **Fig. S1.** Qualitative change in the ADAScog11 scale. Modified Intention-to-Treat Population. **Fig. S2.** Sensitivity analysis. Primary outcome ADAS-Cog11. **Fig. S3-1.** Cerebral perfusion sequencies of two subjects at baseline and at week 48 (worsening). **Fig. S3-2.** Cerebral perfusion sequencies of two subjects at baseline and at week 48 (no changes). **Fig. S3-3.** Cerebral perfusion sequencies of two subjects at baseline and at week 48 (improvement). **Fig. S4.** Percentage of change in hippocampal volume vs. baseline ADAS-Cog11 score. **Fig. S5.** Individual variation in hemoglobin levels. **Fig. S6.** Benefit-risk curves. **Fig. S7.** Individual variation of primary outcome ADAS-Cog11 by stage.

## Data Availability

The datasets used and/or analyzed during the current study are available from the corresponding author on reasonable request.

## Abbreviations

AChEIs: Acetylcholinesterase inhibitors
AD: Alzheimer’s disease
ADAS-Cog11: 11-Item cognitive subscale of the Alzheimer’s Disease Assessment Scale
ADL: Activities of daily living
APOE: Apolipoprotein E
ARIA: Amyloid-related imaging abnormalities
ARIA-E: Amyloid-related imaging abnormalities of edema/effusions
ARIA-H: Amyloid-related imaging abnormalities of microhemorrhages/hemosiderin deposits
CIBIC +: Clinician Interview-Based Impression of Change Incorporating Caregiver Information
CIM: Central of Molecular Immunology
CNS: Central nervous system
COVID: Coronavirus disease
CSF: Cerebrospinal fluid
EPO: Erythropoietin
EPOR: Erythropoietin receptor
eTIV: Estimating the total intracranial volume
FDA: The U.S. Food and Drug Administration
GDS: Global Deterioration Scale
HC: Hippocampal
I L-1β: Interleukin-1 beta
LSM: Least-squares mean
mITT: Modified intention-to-treat population
MoCA: Montreal Cognitive Assessment
MRI: Magnetic resonance imaging
MWPC: Meaningful within patient change
NIA-AA: National Institute on Aging – Alzheimer’s Association
NPI: Neuropsychiatric Inventory
PP: Per protocol population
RPCEC: The Cuban Public Registry of Clinical Trials
SCA2: Spinocerebellar ataxia type 2
SMD: Standard mean difference
SPECT: Single-photon emission computed tomography
TNF-α: Tumor necrosis factor
vMRI: Volumetric MRI
