# Supplementary material for: NeuroEPO plus (NeuralCIM®) in mild-to-moderate Alzheimer’s clinical syndrome: the ATHENEA randomized clinical trial
Source: Alzheimers Res Ther. 2023 Dec 13;15:215. doi: 10.1186/s13195-023-01356-w (PMC10716956; doi:10.1186/s13195-023-01356-w)
Supplement: Supplementary file 1 — Additional file 1: Supplement Methods and Results. Table S1. Secondary outcomes (ADL) from Baseline to 48 Weeks. Table S2. Changes in global cerebral perfusion from baseline to week 48. Table S3. Initial and final hippocampal volume adjusted by eTIV. Table S4. Percentage of change in the hippocampal volume adjusted by eTIV. Table S5. Hemoglobin variation, Bonferroni-corrected (adjusted alpha error level of 0.017). Table S6. Primary outcome ADAS-Cog11 score by stage. Table S7. Clinical response (ADAS-Cog value) stratified by APOE genotype and groups. Fig. S1. Qualitative change in the ADAScog11 scale. Modified Intention-to-Treat Population. Fig. S2. Sensitivity analysis. Primary outcome ADAS-Cog11. Fig. S3-1. Cerebral perfusion sequencies of two subjects at baseline and at week 48 (worsening). Fig. S3-2. Cerebral perfusion sequencies of two subjects at baseline and at week 48 (no changes). Fig. S3-3. Cerebral perfusion sequencies of two subjects at baseline and at week 48 (improvement). Fig. S4. Percentage of change in hippocampal volume vs. baseline ADAS-Cog11 score. Fig. S5. Individual variation in hemoglobin levels. Fig. S6. Benefit-risk curves. Fig. S7. Individual variation of primary outcome ADAS-Cog11 by stage. [file 13195_2023_1356_MOESM1_ESM.docx]

**Supplemental Online Content**

**NeuroEPO plus (NeuralCIM®) in mild-to-moderate Alzheimer’s clinical syndrome: The ATHENEA randomized clinical trial.**

Sosa S, Bringas G, Urrutia N, Peñalver AI, López D, González E, Fernández A, Hernández ZM, Viña A, Peña Y, Batista JF, Valenzuela C, León K, Crombet T, Rodríguez T, Pérez L, ATHENEA Investigators.

Supplement Methods and Results

This supplemental material has been provided by the authors to give readers additional information about their work.

Contents

[**Methods** 3](#_Toc151373474)

[**SPECT acquisition and analysis** 3](#_Toc151373475)

[**MRI acquisition and analysis** 4](#_Toc151373476)

[**Statistical** 7](#_Toc151373477)

[Sample Size Calculation 7](#_Toc151373478)

[Benefit-Risk analysis 9](#_Toc151373479)

[**Results** 10](#_Toc151373480)

[**Tables** 10](#_Toc151373481)

[Table S1 Secondary outcomes (ADL) from Baseline to 48 Weeks. ^*^ 10](#_Toc151373482)

[Table S2 Changes in global cerebral perfusion from baseline to week 48. ^*^ 11](#_Toc151373483)

[Table S3 Initial and final hippocampal volume adjusted by eTIV. ^*^ 11](#_Toc151373484)

[Table S4 Percentage of change in the hippocampal volume adjusted by eTIV. ^*^ 11](#_Toc151373485)

[Table S5. Hemoglobin variation, Bonferroni-corrected (adjusted alpha error level of 0.017). 12](#_Toc151373486)

[Table S6 Primary outcome ADAS-Cog11 score by stage. 12](#_Toc151373487)

[Table S7. Clinical response (ADAS-Cog value) stratified by APOE genotype and groups. 13](#_Toc151373488)

[**Figures** 14](#_Toc151373489)

[**Fig. S1** Qualitative change in the ADAScog11 scale. Modified Intention-to-Treat Population. * 14](#_Toc151373490)

[Fig. S2 Sensitivity analysis. Primary outcome ADAS-Cog11. 15](#_Toc151373491)

[Fig. S3-1 Cerebral perfusion sequencies of two subjects at baseline and at week 48 (worsening). 16](#_Toc151373492)

[Fig. S3-2 Cerebral perfusion sequencies of two subjects at baseline and at week 48 (no changes). 17](#_Toc151373493)

[Fig. S3-3 Cerebral perfusion sequencies of two subjects at baseline and at week 48 (improvement).18](#_Toc151373494)

[Fig. S4 Percentage of change in hippocampal volume vs. baseline ADAS-Cog11 score. ^*^ 19](#_Toc151373495)

[Fig. S5 Individual variation in hemoglobin levels. ^*^ 20](#_Toc151373496)

[Fig. S6 Benefit-risk curves. 21](#_Toc151373497)

[Fig. S7 Individual variation of primary outcome ADAS-Cog11 by stage. ^*^ 22](#_Toc151373498)

[**References** 23](#_Toc151373499)

# **Methods**

## **SPECT acquisition and analysis**

Functional neuroimaging by SPECT was performed on the first 10 patients included in each group. The studies were performed before and 48 weeks after treatment, by qualified personnel (two doctors specializing in nuclear medicine, with more than 15 years of experience in performing and evaluating brain SPECT of subjects with schizophrenia or cognitive impairment or dementia, including clinical Alzheimer's syndrome). For the scintigraphic procedure, the participants were required to abstain from the use of central nervous system (CNS) stimulants or depressants, such as alcohol, tobacco, xanthines, caffeine and psychotropic drugs, for at least 12 hours before the start of the investigation.

SPECT images were acquired 30-60 minutes after intravenous administration of 740 MBq to 925 MBq of 99mTc-ECD (Ethylenecysteine dimer). The patients had their eyes closed and were placed in an environment without auditory or visual stress for five minutes before the injection.

The data acquisition protocol used the following parameters: a window energy of 20% for 99mTc (140 keV) photopeak, a low-energy high resolution (LEHR) collimator at 30 seconds per projection and 120 images in a double-headed gamma camera, NuclineSpirit DH-V (Mediso, Hungary).

The SPECT slices are viewed in the transverse, sagittal, or coronal planes or as three-dimensional (3D) representation. Symmetric bilateral posterior temporal and parietal cortical hypoperfusion with posterior predominance was considered a typical pattern of AD. In severe cases, the hypoperfusion could also be located in the frontal region.

The quantitative representation of the regional perfusion was provided by a colour scale, considering the uptake of radiotracer. Hyperperfusion was represented by colours ranging from red to white and hypoperfusion was represented by colours that range from light green to dark blue to black and suggest a progressive reduction in the brain perfusion.

The changes in the perfusion were evaluated in relation to the baseline study. Assessment of cerebral perfusion were qualitative and semiquantitative, based on color change (see below). The evaluations, both initial and final, were carried out double-blind. The readers were not blinded to visit ID, but they were blinded to treatment group


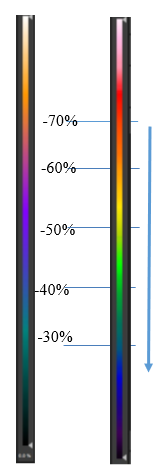


- Significant increase: Increase more than 10% respect to baseline (significant increase in cerebral perfusion).
- Slight increase: Increase of 5-10% in some regions, without variations in others, respect to baseline (non-significant increase in cerebral perfusion).
- No changes: No change from the baseline (no variation in cerebral perfusion).
- Decrease: Decrease more than 5% respect to baseline (worsening in cerebral perfusion)

Decrease perfusion (worsening)

## **MRI acquisition and analysis**

The MRI was acquired using a 3T scanner (Siemens Magnetom Allegra; Siemens Healthcare, Erlangen, Germany) at Cuban Neurosciences Center (CNEURO).

The Dementia Protocol consisted of an axial T1-weighted 3-D scan, sagittal weighted 3-D scan, FLAIR images, high-definition axial T2- weighted scan, axial susceptibility-weighted imaging, axial diffusion (DWI); diffusion gradient encoding, b = 0 and 1000 s/ mm^2^ and 12 directions, and an apparent diffusion coefficient map derived from the diffusion scan.

The pulse sequences used was the Alzheimer's Disease Neuroimaging Initiative (ADNI). Total study time per patient ranged was 38-40 min. We acquired 3D sagittal sequences with axial and coronal reconstruction. In this study, we used the T1-3D MP-RAGE sequence (voxel size [mm3]: 0.8 × 0.8 × 1; time [ms]: TE = 2.6; TR = 2000; TI = 900; duration [min]: 9:19) for automatic segmentation of the hippocampus.

In an international effort to standardize existing protocols in 2014, researchers from the European Alzheimer’s Disease Consortium (EADC) and the ADNI created the EADC-ADNI Harmonized Protocol for Hippocampal Segmentation ([www.hippocampal-protocol.net](http://www.hippocampal-protocol.net)).

For the automatic segmentation of the hippocampus, we used the FreeSurfer 6.0 software, which is available free of cost for Linux and macOS (http://surfer.nmr.mgh. harvard.edu/). This software performs segmentation of brain structures, including the hippocampus, using probability maps. Image processing was performed with a high-performance computer (HPC) system with a peak performance of 4 Tflops. The HPC system includes 5 computing nodes with 2 Intel Xeon CPUs with 24 cores each, for a total of 48 cores per node, with 48 GB of RAM and two 250-GB HDDs each. The system has a 24-TB network access storage system, which stores all the processed data. All images were visually inspected before processing, in accordance with quality control procedures previously described in the literature.

Digital Imaging and Communication in Medicine (DICOM) images were converted to the Neuroimaging Informatics Technology Initiative (NIfTI) format using the multiplatform MRIcroGL software (https:// [www.nitrc.org/plugins/mwiki/index.php/mricrogl:MainPage](http://www.nitrc.org/plugins/mwiki/index.php/mricrogl:MainPage)) and subsequently processed with FreeSurfer 6.0, using the longitudinal pipeline. ^1, 2^

Following the state of the art to performing longitudinal studies, two brain MRIs 12 months apart were acquired. Three steps were used for processing and obtaining the longitudinal results. The first, is a cross-sectional processing; the second, is a base template shaping, and the third is a longitudinal processing using the results of the previous steps.

All hippocampal volumes were adjusted for intracranial volume (ICV). The percent change in hippocampal volume (% change) was calculated:

% change = (final volume – initial volume) x 100

Initial volume

Statistical analysis was performed using the IBM SPSS Statistics software, version 26.0. Contrasts groups were conducted using surface-based linear models, as implemented in the SurfStat toolbox <https://www.math.mcgill.ca/keith/surfstat/>. The model was:

$$Y\sim\mu+Grupo+Grupo*X+e$$

Y: the dependent variable.

Group: fixed main effect.

X: covariate (age, ADAS-Cog11).

e: random error.

For comparison, we used MRI scans of 367 healthy controls (age, ≥ 50 years old) from the free-access Cambridge Centre for Ageing Neuroscience (Cam-CAN) database (<https://camcan-archive.mrc-cbu.cam.ac.uk/dataaccess/>). The z-statistic measured how many standard deviations the sample mean is from the population mean

$$Z=\frac{Y-\overbrace{Y}}{\sigma}$$

The MRI images were subjected to quality control, to detect outliers a quantitative method based on the Grubb statistician was used:

$$G=\frac{max\left| y_{i}-\bar{y} \right|}{s}$$

y_i_ is an outlier if:

$$G>\frac{N-1}{N}\sqrt{\frac{t^{2}(\frac{\alpha}{2N}, N-2)}{N-2+t^{2}(\frac{\alpha}{2N}, N-2)}}$$

The test is calculated iteratively recalculating the value of G.

## **Statistical**

## Sample Size Calculation

The study sample population was calculated using Sample Size Table for Clinical Studies, option Two-Sample t-Test - Unequal Variances:

$$N=\left( \frac{\tau+\varphi}{\varphi} \right)\frac{\left( z_{1-\alpha/2}+z_{1-\beta} \right)^{2}}{\Delta^{2}}+\frac{\left( \tau^{2}+\varphi^{3} \right)z_{1-\alpha/2}^{2}}{2\varphi\left( \tau+\varphi\right)^{2}}$$

$\Delta=\frac{\mu_{2}-\mu_{1}}{\sigma_{1}}$ y $\sigma_{2}^{2}=\tau\sigma_{1}^{2}$

α: 0.025

power 1-β: 0.8

difference in two population means, $\mu_{2}-\mu_{1}$: 2

standard deviation for the placebo group, $\sigma_{1}^{2}$: 2

variance ratio, $\tau={\sigma_{2}^{2}}/{\sigma_{1}^{2}}$: 2

ratio between groups, ϕ: 1

The total was 102 subjects, 30 by groups of treatment. Assuming 30% discontinuation rate, the sample size increased to 114 participants, 38 by group.

It was stablished in the first stage (exploratory study), when the first 20 subjects of each group were included and evaluated after 6 months of treatment:

a) Assess the blind readjustment of the sample size based on the observed variance.

b) To consider postponing the evaluation of the hypothesis until week 48, considering the unavailability of data about the effect of neuroEPO plus at 24 weeks in this disease and that the previous data available on progression in the control group was limited. This analysis was carried out based on the estimation of the probability of success:

1. If the probability of fulfilled the study hypothesis at 24 weeks was small in both dose groups $P\left(\theta>2ptos\right)<0.1$, *(Ɵ* is the final distribution of the difference in the ADAS-cog11 score neuroEPO plus – placebo), the evaluation of the hypothesis was postponed to 48 weeks.
2. If the above condition occurred in only one of the dose groups:

- The inclusion in this group stopped. The treatment scheme was completed until 48 weeks, to the patients already included.
- The patients included in the placebo group, completed 24 weeks of treatment with the dose of NeuroEPO that remained active in the study

During the trial, the stop criterion for unacceptable toxicity (more than 20% of individuals with related serious adverse events.

1. Regarding the assessment of the readjustment of the sample size with the data from the evaluation of the first 60 subjects at 24 weeks, the ratio between the variances was in the considered order (2 units), but the standard deviation (SD) of the control group was greater than that considered in the initial calculation (SD: 2):

- PP analysis: Ratio between the variances of neuroEPO plus groups vs. placebo: 2.1 and 1.8, respectively (SD of the control group: 2.5).
- ITT analysis: Ratio between the variances of neuroEPO plus groups vs. placebo: 1.9 and 1.4, respectively (SD of the control group: 3.4).

It was decided to adjust according to the PP analysis, considering 20% discontinuation rate:

α: 0.025

power 1-β: 0.8

difference in two population means, $\mu_{2}-\mu_{1}$: 2 (hypothesis)

standard deviation for the placebo group, $\sigma_{1}^{2}$: 2.5

variance ratio, $\tau={\sigma_{2}^{2}}/{\sigma_{1}^{2}}$: 2

ratio between groups, ϕ: 1

The adjustment implied a total sample size of 174 subjects (58 per group), which represented an increase of 60 subjects respect to the planned sample size (114 subjects).

1. Regarding to consider postponing the evaluation of the main variable, in both neuroEPO plus groups, the probability of compliance the hypothesis was less than 0.1, therefore the decision was to transfer the evaluation of the hypothesis until 48 weeks.

## Benefit-Risk analysis

Bayes Factor, defined as the likelihood ratio of two hypotheses was used as a measure of Benefit–Risk ratio:

$$Bayes Factor (BF)=\frac{P(data/benefit)}{P(data/risk)}$$

Specifically using Beta distributions for represent and estimate density functions associated to both, in the scenarios planned for Benefit (in terms of ADAS-Cog11) and Risk (in term of serious treatment-related adverse events). Specifically:

Benefit = Increase in the ADAS-Cog11 scale score ≤ 2-3 points respect to the estimated mean in the placebo group.

Risk = Treatment-related serious adverse events.

BF ≥ 1: Evidences in favour to the benefit.

1 < BF ≤ 10^1/2^: Minimal evidence in favour to the benefit.

10^1/2^ < BF ≤ 10: Substantial evidence in favour to the benefit.

10 < BF ≤ 10^2^: Strong evidence in favour to the benefit.

BF > 10^2^: Striking evidence in favour to the benefit.

# **Results**

## **Tables**

### Table S1 Secondary outcomes (ADL) from Baseline to 48 Weeks. ^*^

| **Group** | **0.5 mg** | **1.0 mg** | **Placebo** |
| --- | --- | --- | --- |
| **Barthel Index median ± IR** | **n=50** | **n=49** | **n=49** |
| Initial | Cte=100 | 100.0 ± 0.0 | Cte=100 |
| Week 48 | 100.0 ± 0.0 | 100.0 ± 0.0 | Cte=100 |
| **Independence Index No. (%)** |  |  |  |
| Initial |  |  |  |
| Mild | 0 | 1 (2.0) | 0 |
| Independent | 50 (100.0) | 48 (98.0) | 49 (100.0) |
| Week 48 |  |  |  |
| Mild | 1 (2.0) | 1 (2.0) | 0 |
| Independent | 49 (98.0) | 48 (98.0) | 49 (100.0) |
| **Katz Index No. (%)** |  |  |  |
| Initial |  |  |  |
| A | 49 (98.0) | 49 (100.0) | 49 (100.0) |
| B | 1 (2.0) | 0 | 0 |
| Week 48 |  |  |  |
| A | 48 (96.0) | 49 (100.0) | 49 (100.0) |
| B | 2 (4.0) | 0 | 0 |
| **Lawton Scale median ± IR** |  |  |  |
| Initial | 6.0 ± 3.0 | 6.0 ± 4.0 | 6.0 ± 3.0 |
| Week 48 | 6.0 ± 4.0 | 6.0 ± 4.0 | 5.0 ± 3.0 |
| **Lawton Interpretation No (%)** |  |  |  |
| Initial |  |  |  |
| Serious | 3 (6.0) | 4 (8.2) | 2 (4.1) |
| Moderate | 9 (18.0) | 12 (24.5) | 11 (22.4) |
| Mild | 23 (46.0) | 16 (32.7) | 22 (44.9) |
| Autonomy | 15 (30.0) | 17 (34.7) | 14 (28.6) |
| Week 48 |  |  |  |
| Serious | 3 (6.0) | 4 (8.2) | 8 (16.3) |
| Moderate | 7 (14.0) | 14 (28.6) | 16 (32.7) |
| Mild | 18 (36.0) | 10 (20.4) | 14 (28.6) |
| Autonomy | 22 (44.0) | 21 (42.9) | 11 (22.4) |

^*^ The analysis was performed in the per protocol population, which included subjects who complied with the protocol sufficiently (more than 90% of treatment with efficacy outcomes at baseline and at 48 weeks without any major deviation of protocol) to ensure that these data would be likely to exhibit the effects of treatment according to the underlying scientific model. Subjects were considerate in their randomized group.

ADL, Activities of Daily Living; Cte, constant; No., number; IR, interquartile range.

### Table S2 Changes in global cerebral perfusion from baseline to week 48. ^*^

|  | **NeuroEPO plus 0.5 mg** | **NeuroEPO plus 1.0 mg** | **Placebo** | **p (H_0_)** ^†^ |
| --- | --- | --- | --- | --- |
| **n=25** | **n=11** | **n=5** | **n=9** |
| **Global perfusion No. (%)** | |  |  |  |
| Improvement | 7 (63.6) | 2 (40.0) | 2 (22.2) | 0.345 |
| No change | 3 (27.3) | 2 (40.0) | 5 (55.6) |
| Worsening | 1 (9.1) | 1 (20.0) | 2 (22.2) |

^*^ The analysis was performed in the per protocol population, which included subjects who complied with the protocol sufficiently (more than 90% of treatment with efficacy outcomes at baseline and at 48 weeks without any major deviation of protocol) to ensure that these data would be likely to exhibit the effects of treatment according to the underlying scientific model. Subjects were considerate in their randomized group.

^†^ Bayesian inference. No., number.

### Table S3 Initial and final hippocampal volume adjusted by eTIV. ^*^

|  | **NeuroEPO plus 0.5 mg (n=35)** | **NeuroEPO plus 1.0 mg (n=31)** | **Placebo (n=32)** | **Total** |
| --- | --- | --- | --- | --- |
| **At baseline values mean x10^3^ (CI)** | |  |  |  |
| Left HC volume | 2.04 (1.93 - 2.15) | 2.05 (1.90 - 2.18) | 2.09 (1.96 - 2.21) | 2.06 (1.99 - 2.23) |
| Right HC volume | 2.08 (1.96 - 2.19) | 2.09 (1.93 - 2.25) | 2.13 (2.00 - 2.24) | 2.10 (2.02 - 2.17) |
| Left vs. right^†^ | t(34) = -1.05, *P* <.30 | t(30) = -1.54, *P* <.13 | t(31) = 0.99, *P* <.32 | t(97) = -2.03, *P* <.04 |
| **At 48 Weeks values mean x10^3^ (CI)** | |  |  |  |
| Left HC volume | 1.97 (1.86 - 2.08) | 1.98 (1.84 - 2.13) | 2.00 (1.87 - 2.14) | 1.99 (1.91 - 2.06) |
| Right HC volume | 2.01 (1.89 - 2.14) | 2.03 (1.86 - 1.86) | 2.08 (1.95 - 2.20) | 2.04 (1.96 - 2.12) |
| Left vs. right^†^ | t(34)= -1.17, *P* <.24 | t(30)= -1.57, *P* <.12 | t(31)= -1.82, *P* <.07 | t(97)= -2.61, *P* <.01 |

^*^ The analysis was performed in the per protocol population, which included subjects who complied with the protocol sufficiently (more than 90% of treatment with efficacy outcomes at baseline and at 48 weeks without any major deviation of protocol) to ensure that these data would be likely to exhibit the effects of treatment according to the underlying scientific model. Subjects were considerate in their randomized group.

^†^t-Student (gl).

eTIV, estimated total intracranial volume; HC, hippocampal; CI, confidence interval; vs., versus.

### Table S4 Percentage of change in the hippocampal volume adjusted by eTIV. ^*^

| **% Change**  **Mean (95% CI)** | **NeuroEPO plus 0.5 mg** | **NeuroEPO plus 1.0 mg** | **Placebo** | **ANOVA** |
| --- | --- | --- | --- | --- |
| Left HC | -3.59 (-5.20 -1.98) | -3.14 (-4.43 -1.83) | -4.16 (-5.86 -2.46) | F(2,95)=0.43, p<0.64 |
| Right HC | -3.25 (-4.35 -2.14) | -3.33 (-4.94 -1.71) | -2.47 (-3.71 -1.22) | F(2,95)=0.52, p<0.59 |
| Total | -3.40 (-4.55 -2.25) | -3.26 (-4.54 -1.96) | -3.32 (-4.60 -2.03) | F(2,95)=0.01, p<0.98 |

^*^ The analysis was performed in the per protocol population, which included subjects who complied with the protocol sufficiently (more than 90% of treatment with efficacy outcomes at baseline and at 48 weeks without any major deviation of protocol) to ensure that these data would be likely to exhibit the effects of treatment according to the underlying scientific model. Subjects were considerate in their randomized group.

eTIV, estimated total intracranial volume; HC, hippocampal; CI, confidence interval

### Table S5. Hemoglobin variation, Bonferroni-corrected (adjusted alpha error level of 0.017).

|  | | | **NE 0.5 mg** | **NE 1.0 mg** | **Placebo** | **p (Kruskal-Wallis)** |
| --- | --- | --- | --- | --- | --- | --- |
| Hemoglobin | **Time (weeks)** | | **N=50** | **N=42** | **N=43** |  |
|  | 0 | Median (IR) | 13.2 (1.4) | 13.0 (1.5) | 13.2 (2.3) | 0.558 |
|  |  | (min; max) | (9.9; 16.5) | (11.4; 15.2) | (11.7; 16.8) |  |
|  | 24 | Median (IR) | 13.1 (1.6) | 13.0 (1.2) | 13.5 (1.7) | 0.078 |
|  |  | (min; max) | (10.2; 16.7) | (9.1; 14.8) | (10.8; 15.0) |  |
|  | 48 | Median (IR) | 12.6 (1.5) | 12.5 (1.4) | 12.9 (1.9) | 0.202 |
|  |  | (min; max) | (9.0; 15.8) | (9.8; 14.7) | (11.5; 15.6) |  |
|  | p (Wilcoxon) | | 0.022 | 0.001 | 0.236 |  |

IR, interquartile range; min., minimum; max., maximum.

^*^ The analysis was performed in the per protocol population, which included subjects who complied with the protocol sufficiently (more than 90% of treatment with efficacy outcomes at baseline and at 48 weeks without any major deviation of protocol) to ensure that these data would be likely to exhibit the effects of treatment according to the underlying scientific model. Subjects were considerate in their randomized group.

### Table S6 Primary outcome ADAS-Cog11 score by stage.

| **Primary outcome** | | **NE 0.5 mg** | **NE 1.0 mg** | **Placebo** | **p (Kruskal-Wallis)** |
| --- | --- | --- | --- | --- | --- |
| Change from baseline to 48 weeks in ADAS-Cog11 score | |  |  |  |  |
| No. of participants evaluated | |  |  |  |  |
| Mild | Mean ± SD | -4.6 ± 3.3 | -4.7 ± 4.6 | 4.4 ± 4.4 |  |
|  | Median ± IR | -4.0 ± 3.0 | -5.0 ± 5.0 | 3.0 ± 6.0 | 0.000 |
|  | Min; Max | (-17; 0) | (-12; 13) | (-4; 16) | (vs. placebo) |
|  | Diff. vs. placebo | 7.0 (1.1) | 8.0 (1.4) |  | |
|  | CI 95% Diff. | (4.8; 9.2) | (5.3; 10.7) |  |  |
|  | $P\left(\theta>2ptos\right)$ | 1.000 | 1.000 |  |  |
|  | $P\left(\theta>3ptos\right)$ | 1.000 | 1.000 |  |  |
| No. of participants evaluated | | 16 | 20 | 13 |  |
| Moderate | Mean ± SD | -3.0 ± 4.4 | -3.9 ± 5.1 | 5.5 ± 2.8 |  |
|  | Median ± IR | -3.0 ± 6.0 | -4.0 ± 7.0 | 6.0 ± 4.0 | 0.000 |
|  | Min; Max | (-12; 6) | (-14; 7) | (0; 9) | (vs. placebo) |
|  | Diff. vs. placebo | 9.0 (2.0) | 10.0 (2.0) |  | |
|  | CI 95% Diff. | (5.0; 13.0) | (6.0; 14.0) |  |  |
|  | $P\left(\theta>2ptos\right)$ | 1.000 | 1.000 |  |  |
|  | $P\left(\theta>3ptos\right)$ | 0.998 | 1.000 |  |  |

^*^ The analysis was performed in the per protocol population, which included subjects who complied with the protocol sufficiently (more than 90% of treatment with efficacy outcomes at baseline and at 48 weeks without any major deviation of protocol) to ensure that these data would be likely to exhibit the effects of treatment according to the underlying scientific model. Subjects were considerate in their randomized group.

NE, neuroEPO plus; SD, standard deviation; IR, interquartile range; min., minimum; max., maximum; diff., difference; vs., versus.

### Table S7. Clinical response (ADAS-Cog value) stratified by APOE genotype and groups.

| **Clinical response**  **No. (%)** | **NE plus 0.5 mg**  **N=16** | **NE plus 1.0 mg**  **N=16** | **Placebo**  **N=12** | **Total**  **N=44** |
| --- | --- | --- | --- | --- |
| APOE ε4 carriers |  |  |  |  |
| Improvement | 10 (62.5%) | 11 (69%) | 1 (8.3%) | 22 (50%) |
| No change | 6 (37.5%) | 3 (19%) | 2 (16.7%) | 11 (25%) |
| Worsening | 0 | 0 | 9 (75%) | 9 (20.5%) |
| Missing data* | 0 | 2 (12%) | 0 | 2 (4.5%) |
| APOE ε4 non-carriers | N=13 | N=14 | N=22 | N=49 |
| Improvement | 5 (38%) | 11 (78%) | 0 | 16 (33%) |
| No change | 7 (54%) | 3 (22%) | 4 (18%) | 14 (28%) |
| Worsening | 1 (8%) | 0 | 18 (82%) | 19 (39%) |

*Missing data: Two patients who did not have the primary endpoint (ADAS-Cog) at week 48, because they abandoned the trial.

APOE ε4 carriers: APOE ε3ε4 (35 patients), APOE ε4ε4 (9 patients); APOE ε4 non-carriers: APOE ε2ε3 (5 patients), APOE ε3ε3 (44 patients).

ADAS-Cog11, scores on the 11-item cognitive subscale of the Alzheimer’s Disease Assessment Scale range from 0 to 70, with higher scores indicating greater impairment (scores were adjusted for age and formal education); NE, neuroEPO plus; No., number.

## **Figures**

### **Fig. S1** Qualitative change in the ADAScog11 scale. Modified Intention-to-Treat Population. *

^*^The analysis was performed in the intention-to-treat-worst scenario, increase of 10 units at week 48 in the ADAS-Cog11 score (ADAS-Cog11, scores on the 11-item cognitive subscale of the Alzheimer’s Disease Assessment Scale range from 0 to 70, with higher scores indicating greater impairment (scores were adjusted for age and formal education). Stabilized, proportion of improvement + no change. CI, confidence interval. **p=0.000


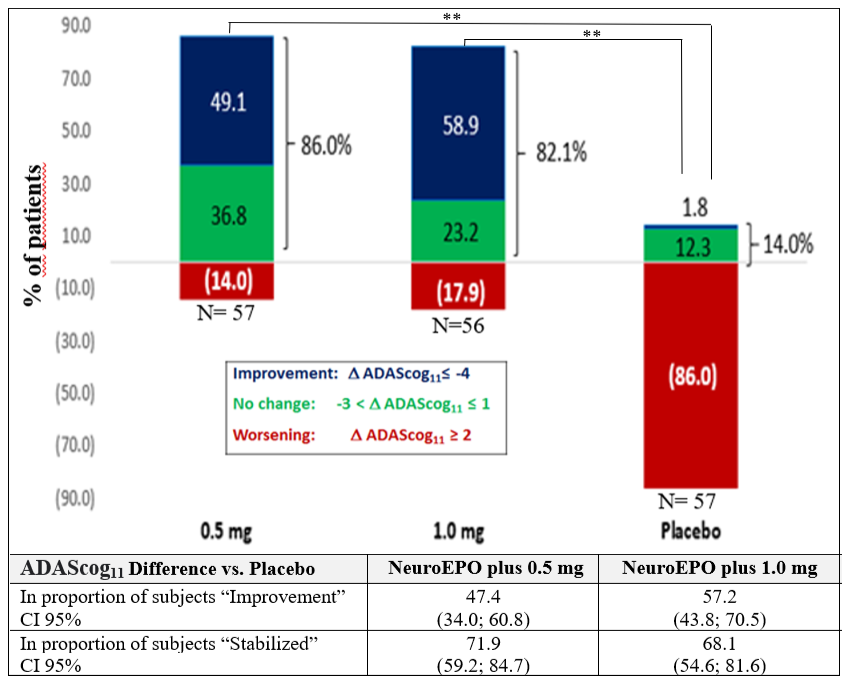


### Fig. S2 Sensitivity analysis. Primary outcome ADAS-Cog11.

PP Per protocol, subjects who complied with the protocol sufficiently (more than 90% of treatment with efficacy outcomes at baseline and at 48 weeks without any major deviation of protocol) to ensure that these data would be likely to exhibit the effects of treatment according to the underlying scientific model. Subjects were considerate in their randomized group.

ITT Intention-to-treat-worst scenario, increase of 10 units at week 48 in the ADAS-Cog11 score.

ADAS-Cog11, scores on the 11-item cognitive subscale of the Alzheimer’s Disease Assessment Scale range from 0 to 70, with higher scores indicating greater impairment (scores were adjusted for age and formal education).


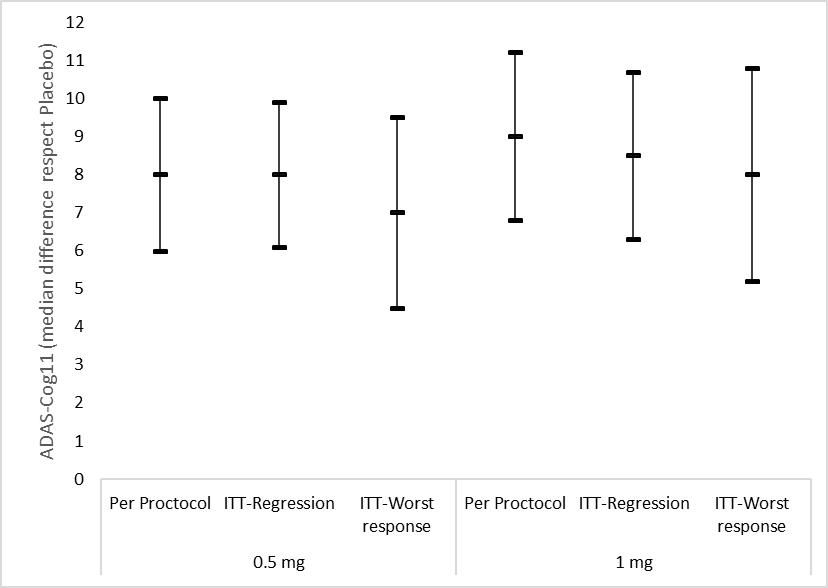


Per Protocol

Per Protocol

### Fig. S3-1 Cerebral perfusion sequencies of two subjects at baseline and at week 48 (worsening).

**Panel A**: Brain perfusion SPECT (cross-sectional, sagittal and coronal sections) performed after intravenous administration of 1130 MBq of 99mTc-ECD. A 76-year-old-female subject enrolled in placebo group. Computed Axial Tomography reported moderate generalized cortical atrophy with ventricular asymmetry. **Before**) Study at baseline. **After**) Study carried out at 48 weeks.

**Panel B**: Brain perfusion SPECT (cross-sectional, sagittal, coronal and 3D views) performed after intravenous administration of 1107 MBq of 99mTC-ECD. A 75-year-old female subject enrolled in the neuroEPO plus 0.5 mg group. Computed Axial Tomography did not report alterations. **Before**) Study at baseline. **After**) Study carried out at 48 weeks.


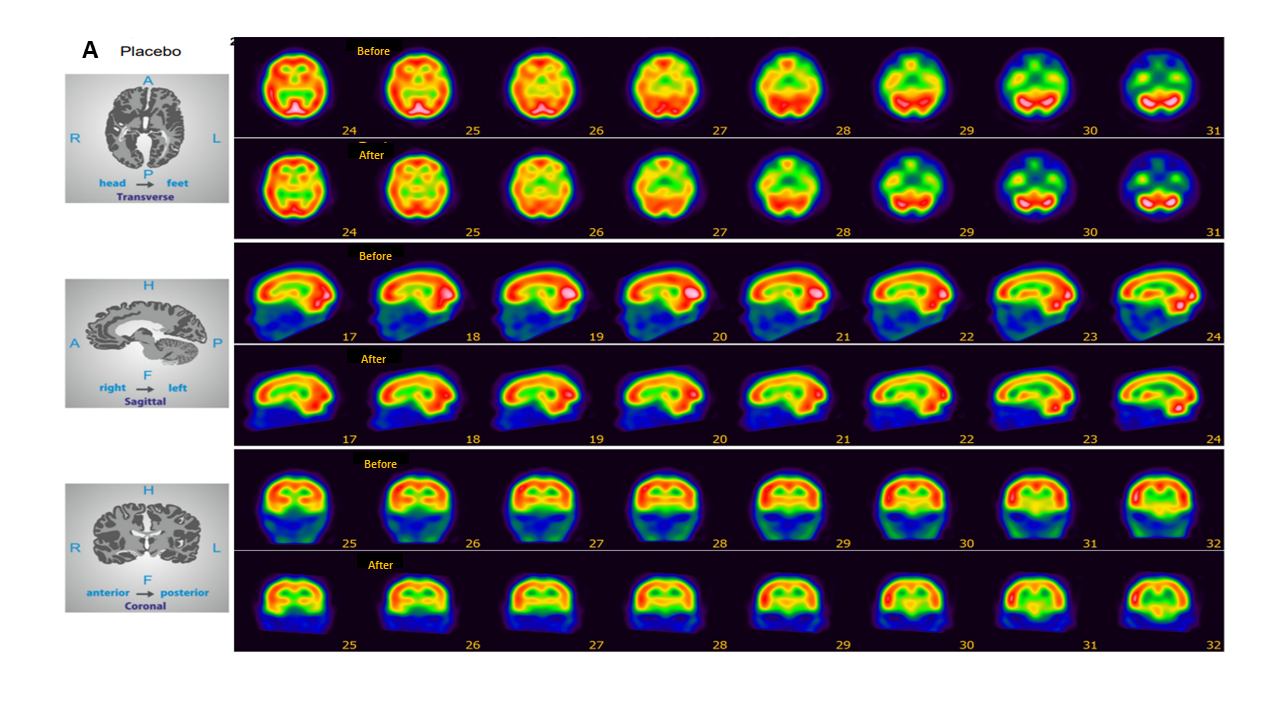


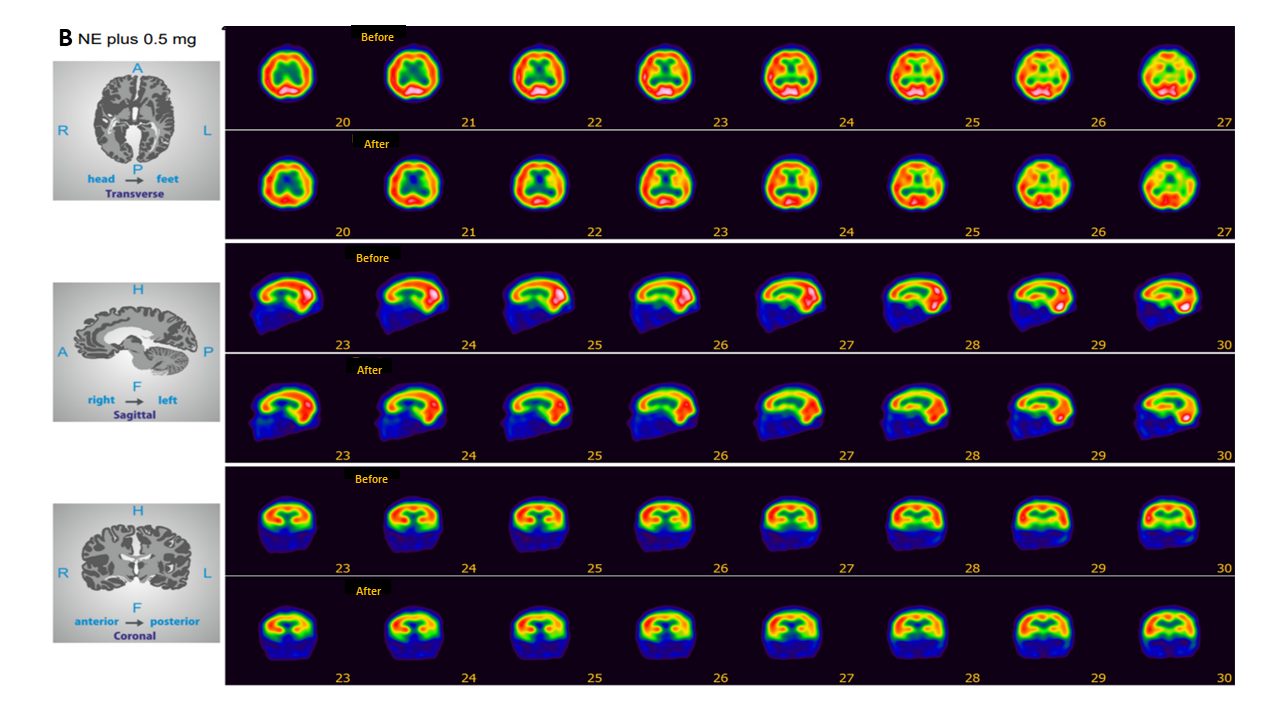


### Fig. S3-2 Cerebral perfusion sequencies of two subjects at baseline and at week 48 (no changes).

**Panel A**: Brain perfusion SPECT (cross-sectional, sagittal and coronal sections) performed after intravenous administration of 1130 MBq of 99mTc-ECD. A 69-year-old-female subject enrolled in placebo group. Computed Axial Tomography reported moderate generalized cortical atrophy with ventricular asymmetry. **Before**) Study at baseline. **After**) Study carried out at 48 weeks.

**Panel B**: Brain perfusion SPECT (cross-sectional, sagittal, coronal and 3D views) performed after intravenous administration of 1107 MBq of 99mTC-ECD. A 66-year-old female subject enrolled in the neuroEPO plus 0.5 mg group. Computed Axial Tomography did not report alterations. **Before**) Study at baseline. **After**) Study carried out at 48 weeks.


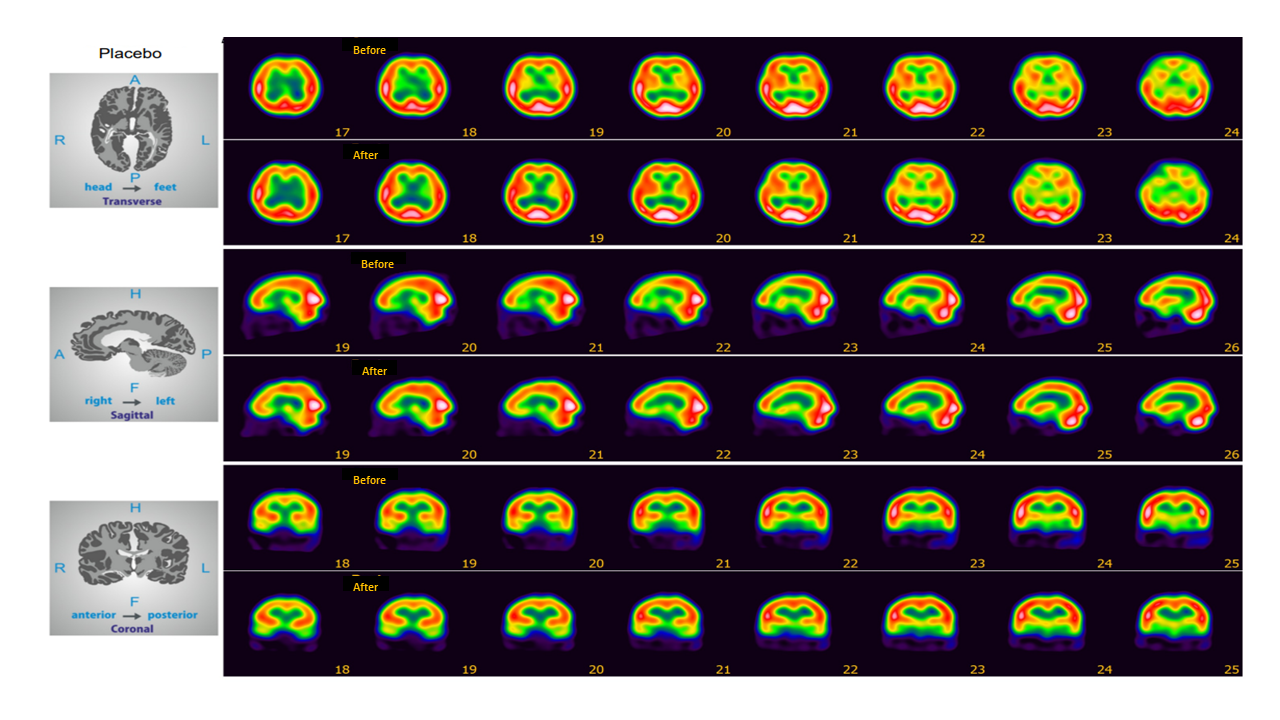


**A**


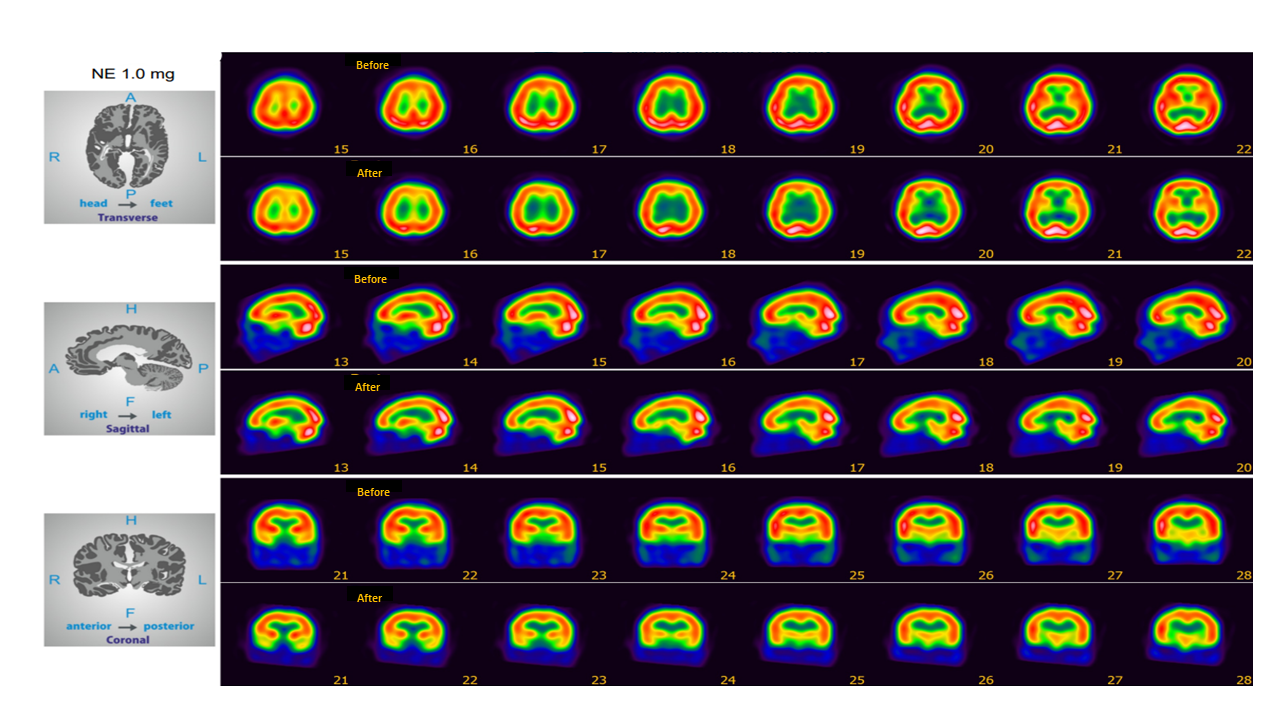


**B**

### Fig. S3-3 Cerebral perfusion sequencies of two subjects at baseline and at week 48 (improvement).

**Panel A**: Brain perfusion SPECT (cross-sectional, sagittal and coronal sections) performed after intravenous administration of 1130 MBq of 99mTc-ECD. A 71-year-old-male subject enrolled in placebo group. Computed Axial Tomography reported moderate generalized cortical atrophy with ventricular asymmetry. **Before**) Study at baseline. **After**) Study carried out at 48 weeks.

**Panel B**: Brain perfusion SPECT (cross-sectional, sagittal, coronal and 3D views) performed after intravenous administration of 1107 MBq of 99mTC-ECD. A 74-year-old male subject enrolled in the neuroEPO plus 0.5 mg group. Computed Axial Tomography did not report alterations. **Before**) Study at baseline. **After**) Study carried out at 48 weeks.


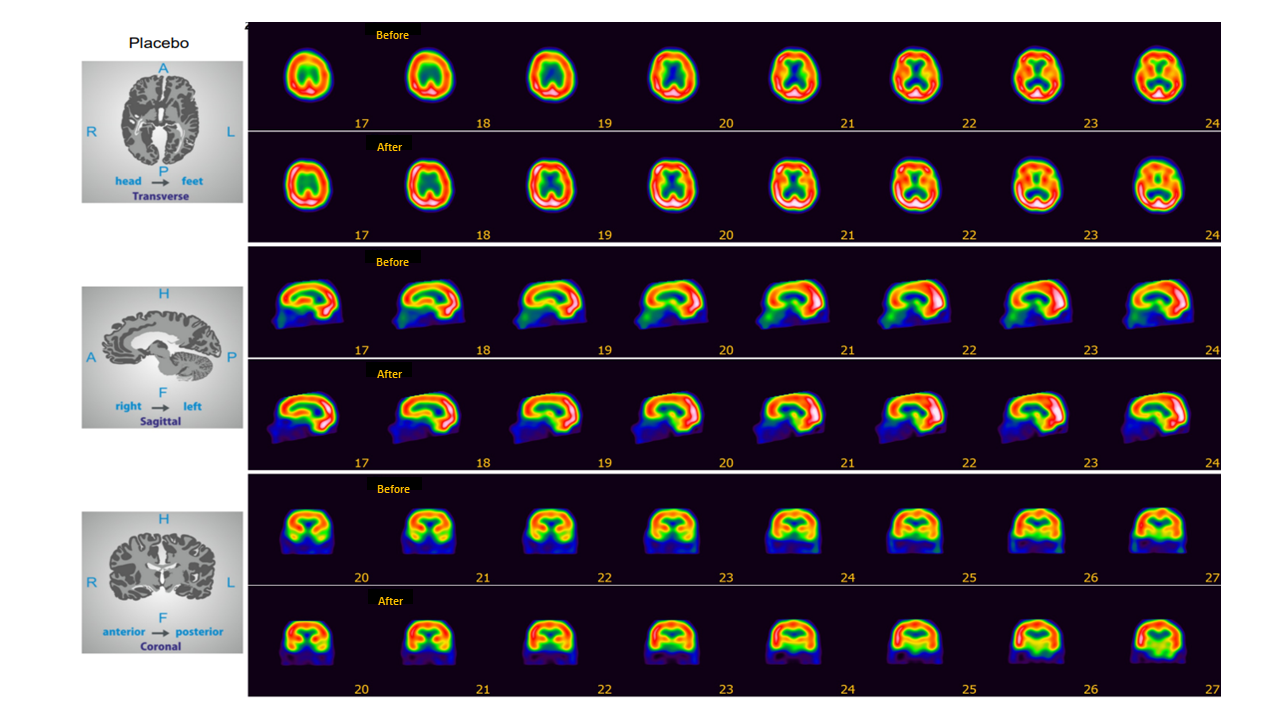


**B**

**A**


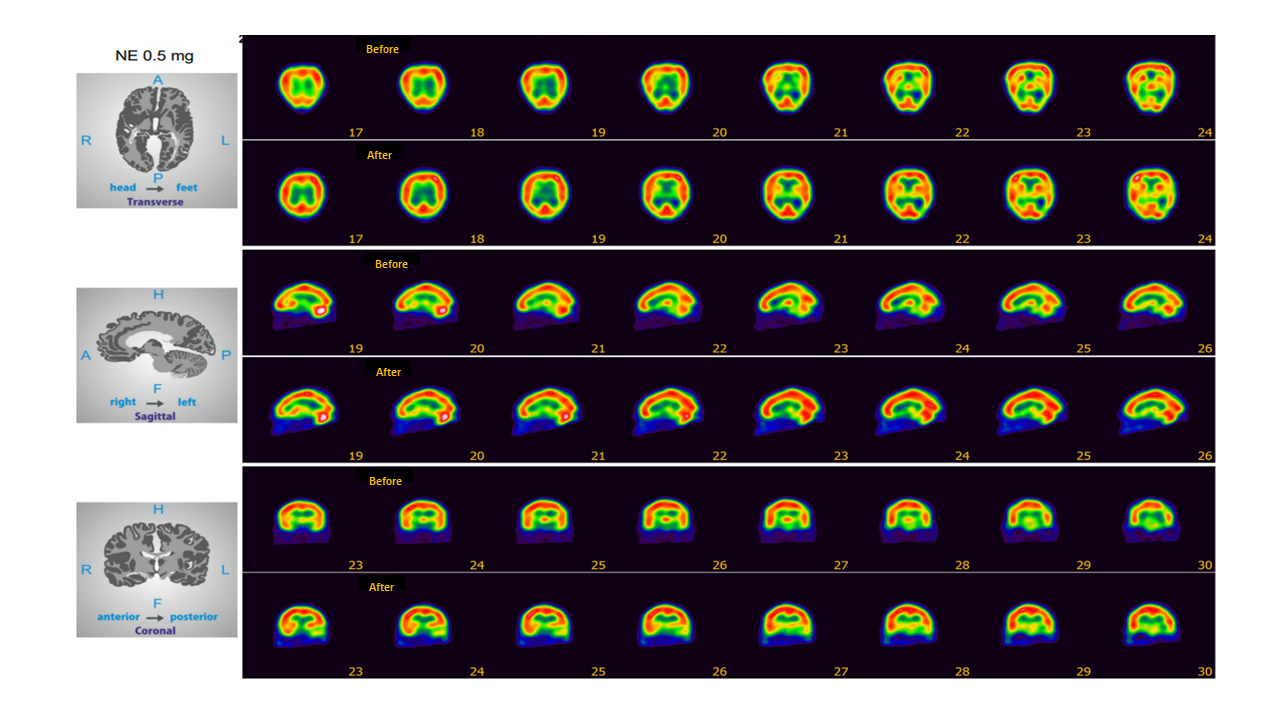


### Fig. S4 Percentage of change in hippocampal volume vs. baseline ADAS-Cog11 score. ^*^

^*^ The analysis was performed in the per protocol population, which included subjects who complied with the protocol sufficiently (more than 90% of treatment with efficacy outcomes at baseline and at 48 weeks without any major deviation of protocol) to ensure that these data would be likely to exhibit the effects of treatment according to the underlying scientific model. Subjects were considerate in their randomized group.

A: NeuroEPO plus 0.5 mg; B: NeuroEPO plus 1.0 mg; C: placebo.

Vol., volume; ADAS-Cog11, 11-item cognitive subscale of the Alzheimer’s Disease Assessment Scale (ADAS-Cog11, with scores ranging from 0 to 70 and higher scores indicating greater impairment).


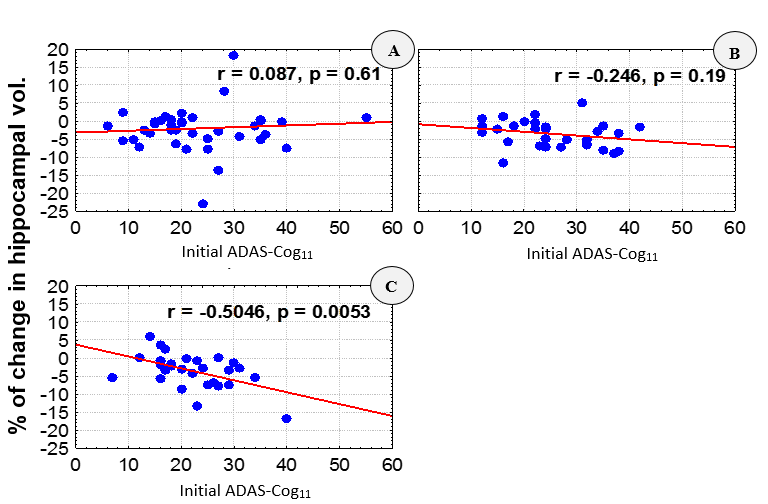


### Fig. S5 Individual variation in hemoglobin levels. ^*^

^*^ The analysis was performed in the per protocol population, which included subjects who complied with the protocol sufficiently (more than 90% of treatment with efficacy outcomes at baseline and at 48 weeks without any major deviation of protocol) to ensure that these data would be likely to exhibit the effects of treatment according to the underlying scientific model. Subjects were considerate in their randomized group.


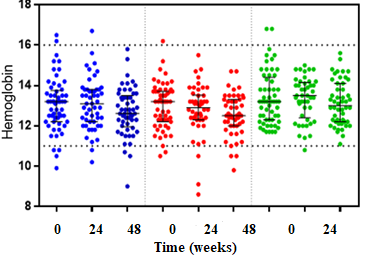


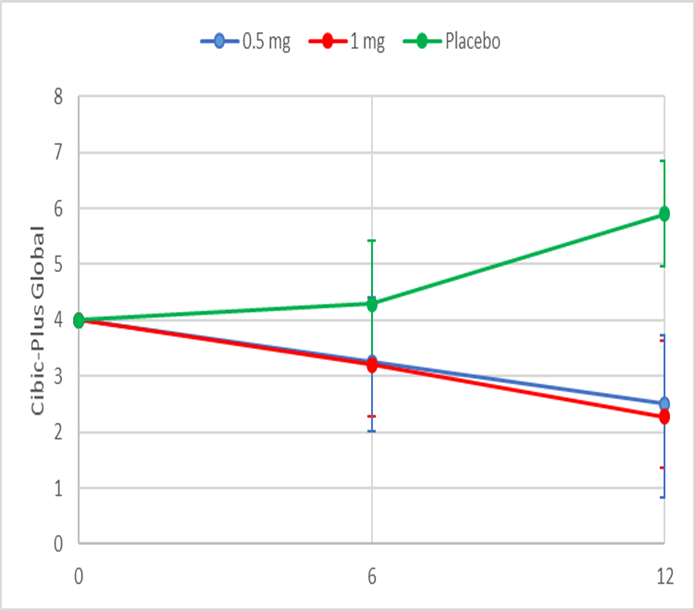


### Fig. S6 Benefit-risk curves.

**Panels A, C, E**: The analysis was performed in the per protocol population, which included subjects who complied with the protocol sufficiently (more than 90% of treatment with efficacy outcomes at baseline and at 48 weeks without any major deviation of protocol) to ensure that these data would be likely to exhibit the effects of treatment according to the underlying scientific model. Subjects were considerate in their randomized group.

**Panels B, D, F**: The analysis was performed in the modified intention-to-treat population (worst scenario, increase by 10 units at week 48 in ADAS-Cog11 scores), which included participants with at least one dose of neuroEPO plus or placebo and a baseline measurement based on randomized treatment.

**Panels A, B**: NeuroEPO plus 0.5 mg.

**Panels C, D**: NeuroEPO plus 1.0 mg.

**Panels E, F**: Placebo.

Benefit = Increase in the ADAS-Cog11 scale score ≤ 2-3 points respect to the estimated mean in the placebo group.

Risk = Treatment-related serious adverse events.

### Fig. S7 Individual variation of primary outcome ADAS-Cog11 by stage. ^*^

^*^ The analysis was performed in the per protocol population, which included subjects who complied with the protocol sufficiently (more than 90% of treatment with efficacy outcomes at baseline and at 48 weeks without any major deviation of protocol) to ensure that these data would be likely to exhibit the effects of treatment according to the underlying scientific model. Subjects were considerate in their randomized group.

Panels show the estimated median change from baseline to week 48 in scores on the 11-item cognitive subscale of the Alzheimer’s Disease Assessment Scale (ADAS-Cog11, with scores ranging from 0 to 70 and higher scores indicating greater impairment). **Panel A**: mild stage and **B**: moderate stage. NE, neuroEPO plus. ** < .005

**
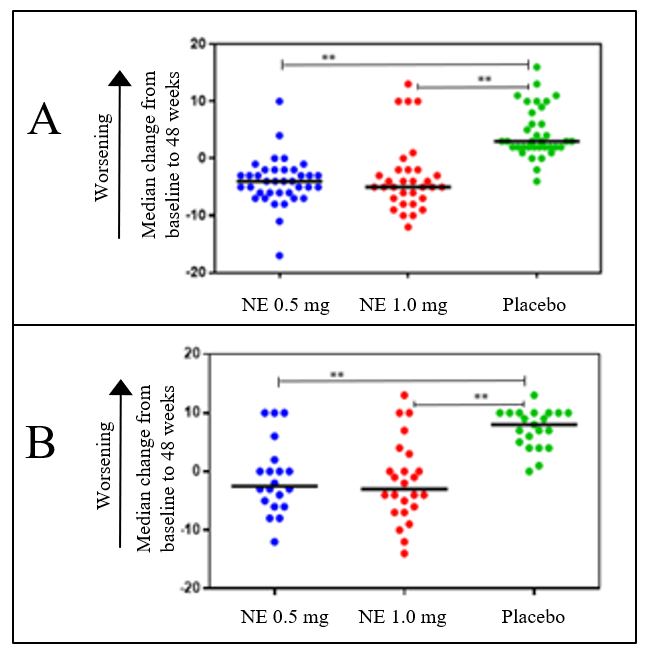
**

# **References**

1. Viña A, González E, Rodríguez I, Pérez L, Rodríguez T. Longitudinal MRI processing to document the effect of drugs in clinical trials. Longitudinal MRI processing to document the effect of drugs in clinical trials. RIELAC. 2021 Dec;42(3):71-81.
2. Reuter M, Rosas HD, Fischl B. Longitudinal FreeSurfer for reliable imaging biomarkers. NIBAD'12. 2012 Oct 5:275.
